# Comparison of Power Training vs Traditional Strength Training on Physical Function in Older Adults: A Systematic Review and Meta-analysis

**DOI:** 10.1001/jamanetworkopen.2022.11623

**Published:** 2022-05-11

**Authors:** Anoop T. Balachandran, James Steele, Daniel Angielczyk, Mark Belio, Brad J. Schoenfeld, Norberto Quiles, Nicole Askin, Ahmed M. Abou-Setta

**Affiliations:** 1 Department of Family, Nutrition, and Exercise Sciences, Queens College, The City University of New York, Flushing, New York; 2 School of Sport, Health, and Social Sciences, Solent University, Southampton, United Kingdom; 3 Department of Exercise and Sport Science, University of North Carolina at Chapel Hill; 4 Health Sciences Department, The City University of New York Lehman College, Bronx, New York; 5 Neil John Maclean Health Sciences Library, University of Manitoba, Winnipeg, Manitoba, Canada; 6 George and Fay Yee Center for Healthcare Innovation, Winnipeg Regional Health Authority, University of Manitoba, Winnipeg, Manitoba, Canada

## Abstract

**Importance:**

Strength training exercise is recommended for improving physical function in older adults. However, whether strength training (lifting and lowering weights under control) and power training (PT) (lifting weights fast and lowering under control) are associated with improved physical function in older adults is not clear.

**Objective:**

To evaluate whether PT vs traditional strength training is associated with physical function improvement in older adults.

**Data Sources:**

Systematic searches of MEDLINE, Embase, Cochrane Central, CINAHL, PsycInfo, PEDro, and SPORTDiscus were conducted from database inception to October 20, 2021.

**Study Selection:**

Randomized clinical trials (RCTs) that compared strength training with instructions to move the weight as fast as possible in the lifting phase with traditional strength training in healthy, community-living older adults (age ≥60 years).

**Data Extraction and Synthesis:**

Two authors independently selected trials, extracted data, assessed the risk of bias using the Cochrane risk-of-bias tool 2, and assessed the certainty of the evidence using the Grading of Recommendations, Assessment, Development and Evaluation approach. Summary effect size measures were calculated using a multilevel random-effects model with cluster robust variance estimation and are reported as standardized mean differences (SMDs). Reporting followed the Preferred Reporting Items for Systematic Reviews and Meta-analyses guideline.

**Main Outcomes and Measures:**

Primary outcomes included physical function and self-reported physical function. Secondary outcomes included power, strength, muscle mass, walk speed, balance, and adverse effects.

**Results:**

A total of 20 RCTs enrolling 566 community-living older adults (mean [SD] age, 70.1 [4.8] years; 368 [65%] women) were included. For the primary outcomes, PT was associated with an improvement in physical function with low-certainty evidence in 13 RCTs (n = 383) (SMD, 0.30; 95% CI, 0.05-0.54) and self-reported function with low-certainty evidence in 3 RCTs (n = 85) (SMD, 0.38; 95% CI, –0.62 to 1.37). The evidence was downgraded by 2 levels for high risk of bias and imprecision for physical function and very serious imprecision for self-reported physical function.

**Conclusions and Relevance:**

In this systematic review and meta-analysis, PT was associated with a modest improvement in physical function compared with traditional strength training in healthy, community-living older adults. However, high-quality, larger RCTs are required to draw more definitive conclusions.

## Key Points

**Question:**

Is power training associated with an improvement in physical function compared with traditional strength training in community-living older adults?

**Finding:**

In this systematic review and meta-analysis of 20 randomized clinical trials enrolling 566 older adults, low-certainty evidence showed improvement in physical function and self-reported function with power training. Power training was associated with an improvement in physical function in 13 RCTs and self-reported physical function in 3 RCTs.

**Meaning:**

The findings of this study suggest that power training may be associated with a modest improvement in physical function compared with traditional strength training in healthy, community-living older adults.

## Teaser

This systematic review and meta-analysis compares strength training vs power training for improving physical function in adults aged 60 years and older.

## Introduction

Aging is associated with a progressive decrease in physical function, loss of physical independence, an increased probability of falls, and reduced quality of life.^1,2,3^ Maintaining function well during late life is as important as prolonging life expectancy in older adults.^4^ Considering that the global aging population is projected to double in number to approximately 1.5 billion by 2050, preserving physical function is a major public health concern.^5,6^ Despite the potential public health impact, few interventions exist to slow the decrease in physical function. Physical activity, and in particular strength training, is a major strategy to prevent or delay mobility disability in older adults.

Strength training is recommended in older adults to improve physical function.^7,8,9,10^ However, muscle power has emerged as an important factor in physical function. During the aging process, muscle power decreases at a faster rate than strength,^11^ and several reports suggest that muscle power is more highly correlated with physical function than strength or muscle mass.^12,13,14^ In contrast to traditional strength training, power training (PT) or high-velocity resistance training involves moving the resistance at higher velocities during the lifting (concentric) phase, followed by a controlled lowering (eccentric) phase.

Previous systematic reviews and meta-analyses have reported that PT is more beneficial than traditional resistance training for improving physical function.^15,16^ However, those reviews included studies using plyometric exercises, such as depth jumps and countermovement jumps.^17,18^ Unlike PT that uses slow, controlled eccentrics, plyometric training uses rapid eccentric movement immediately followed by a rapid concentric contraction to initiate the stretch-shortening cycle.^19^ Furthermore, previous analyses included studies with multiple cointerventions across groups. For example, the largest study used standing functional exercises for PT, and the control group used seated exercises.^20^ The inclusion of multiple interventions makes it impossible to assess the specific outcomes associated with PT. Therefore, the actual utility of PT per se in functional outcomes in older adults is still uncertain.

Considering the limitations of the literature, we systematically reviewed PT vs traditional strength training and measures of physical function in community-living older adults. Moreover, we carried out a multilevel meta-analysis to quantify the magnitude of the outcomes.

## Methods

### Data Sources

The review protocol was prospectively registered on PROSPERO (CRD42020149015) and our findings are reported according to the Preferred Reporting Items for Systematic Reviews and Meta-analyses (PRISMA) guideline. A research librarian conducted a systematic search of the following 7 databases: MEDLINE (Ovid), Embase (Ovid), Cochrane Central (Wiley), CINAHL (Ebsco), PsycInfo, PEDro, and SPORTDiscus in October 1, 2019, with no date limits. We also conducted a forward search of included studies until October 10, 2021, using Google Scholar. We subsequently updated our search until October 20, 2021. Citation management was performed using Endnote X9 (Clarivate).

### Eligibility Criteria

We included randomized clinical trials (RCTs) lasting at least 6 weeks. Populations included healthy, community-living older adults, with a mean age of at least 60 years. In addition to age, we extracted data on sex but no other demographic characteristics because they were not relevant to the study outcomes. We only considered interventions that included resistance training (eg, machines, free weights, elastic tubing, weighted vests, or cycle ergometry) with instructions to move the resistance as fast as possible in the concentric phase. For the comparator group, we excluded nonstrength training control groups.

### Data Selection and Extraction

Two of us (A.T.B., D.A.) independently screened titles and abstracts and then independently read full texts to confirm eligibility. Any disagreements were resolved by consensus and by another one of us (A.M.A.-S.).

Two of us (A.T.B., J.S.) independently piloted a data collection form and then independently extracted outcome data. Extracted data were compared by 2 of us (B.J.S., N.Q.), and any discrepancies were resolved through discussion. If data were only presented graphically, values were estimated from figures using WebPlotDigitizer, version 4.3.^21^ If data were not available, we attempted to contact study authors.

### Outcomes

The preregistered primary outcomes were physical function and self-reported physical function: physical function included both composite outcomes, (eg, Short Physical Performance Battery Score, Continuous Scale Physical Functional Performance) and power-related single measures of function (chair stand, Get Up & Go, stair climb). These measures are well validated and widely used to assess function in older adults.^22^ Self-reported physical function was determined by validated questionnaires. The secondary outcomes included lower or upper body strength and power; muscle mass, fat-free mass, or muscle thickness; usual and fast gait speed; static, dynamic, or reactive balance; and adverse events.

### Assessment of Risk of Bias

Two reviewers (A.T.B., M.B.) independently rated the risk of bias of the RCTs using the revised Cochrane risk of bias, version 2 (RoB 2) tool.^23^ The assignment or intention to treat was the outcome of interest. Disagreements were resolved by consensus. We contacted authors when information was not reported in the article and/or needed clarification.

### Certainty of Evidence

Two of us (A.T.B., M.B.) independently rated the certainty for each comparison and outcome as high, moderate, low, or very low, based on the Grading of Recommendations, Assessment, Development and Evaluation (GRADE) method.^24^ We used a minimally contextualized approach, with a null effect as the threshold of importance to make judgments for the primary outcomes and small effect size as the threshold for secondary outcomes.^25^

### Statistical Analysis

Standardized effect sizes were calculated for pre-post control trial designs with Hedges *g* value using the pooled group baseline SD as the numerator.^26^ Pre-post correlations for measures are often not reported in original studies; thus, we examined a range of values to inspect the sensitivity of our results. Herein, we report outcomes for *r* = 0.7 but include outcomes for both *r* = 0.5 and *r* = 0.9 in eTable 6 in the Supplement. Standardized effect sizes were interpreted per Cohen thresholds^27^: trivial (<0.2), small (0.2 to <0.5), moderate (0.5 to <0.8), and large (≥0.8). Quantitative synthesis of data was performed with the metafor package in R, version 4.0.2.^28^

Because there was a nested structure to the effect sizes calculated from the RCTs included (ie, multiple outcomes nested within groups and nested within studies), multilevel mixed-effects meta-analyses were performed. Cluster robust point estimates and precision of those estimates using 95% CIs were produced, weighted by inverse sampling variance to account for the within- and between-study variance (τ^2^). Restricted maximum likelihood estimation was used in all models. Two main models were produced for both preregistered main outcomes (physical function and self-reported physical function). For the preregistered models, we included composite outcomes (eg, Short Physical Performance Battery Score) preferentially if reported, and if they were not reported, we included all other power-based outcomes noted (eg, chair rise, up and go tests, stair climb) but also explored the sensitivity of results to the inclusion of these individually.

In addition to the main models, we explored moderators for the preregistered physical function outcomes. Subgroup and meta-regression analyses included mean sample age, mean sample body mass index (calculated as weight in kilograms divided by height in meters squared), proportion of samples that were male, baseline functional status (low vs high), duration of intervention, frequency of intervention, and the relative loads used during the intervention. In addition, we explored all physical function outcomes combined (and moderators), all strength outcomes, all power outcomes, all muscle mass and size outcomes, all gait outcomes separately from other physical function, and all balance outcomes. We also examined adherence proportions and a Poisson regression model for adverse event count data (per 1000 person-sessions).

For assessment of heterogeneity, *Q* and *I*^2^ statistics are reported. An *I*^2^ value greater than 50% represented substantial heterogeneity for continuous outcomes. The risk of small-study bias was examined visually through contour-enhanced funnel plots. Influence analyses was performed examining Cook distances for the main models and if there was evidence of influential effect sizes (Cook D approximately 1.0 or, more conservatively, approximately 4/K, where K is the number of studies) and if necessary, models were rerun dropping that outcome to explore the sensitivity of results. All analysis code, data, sensitivity analyses, and data visualizations are available.^29^

## Results

**Figure 1.**
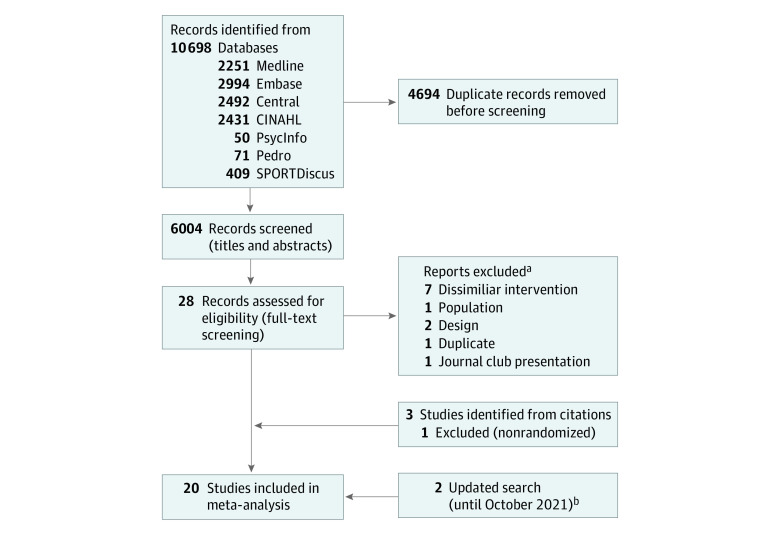
Flow Diagram of Trial Identification and Selection ^a^Reports excluded are reported in eTable 1 in the Supplement. ^b^Flowchart for updated search until October 20, 2021, in eFigure 1 in the Supplement.

From 10 698 citations identified by our search strategy, we included 20 RCTs (Figure 1). The search strategy for all databases is presented in eAppendix 1 in the Supplement and excluded studies with reasons are given in eTable 1 in the Supplement. We subsequently updated our search until October 20, 2021, and included 2 additional trials (eFigure 1 in the Supplement).

### Characteristics of Included Studies

All RCTs used a parallel group design. The sample sizes were relatively small, with a median of 12 (range, 8-20) participants in PT and 13 (range, 7-25) in traditional strength training.

#### Population

Twenty trials enrolling 566 participants from 6 countries were included (Table). The mean (SD age of the participants was 70.1 (4.8) years, most participants were women (368 [65%] vs 198 [35%] men) and body mass index ranged from 21 to 30.37, with most participants classified as overweight. Most of the studies (14 of 20 [70%]) enrolled community-living older adults who were well functioning, and 6 RCTs recruited older adults with low to moderate functioning.^30,33,39,50,51^

**Table.** Physical Function Measures in Studies Included in the Systematic Review

Source	Baseline, No.		Post, No.		Age (mean), y	Male, %	Duration	Freqency	Sets	Repetitions	Sets	Repetitions	Intensity, %		Concentric velocity		Primary outcomes^a^^,^^b^
	Int	Con	Int	Con					Int	Int	Con	Con	Int	Con	Int	Con	
Fielding et al,^30^ 2002	15	15	12	13	73	0	12	3	3	8	3	8	70	70	1	3	None
Bottaro et al,^31^ 2007	12	12	11	9	66.45	100	10	2	3	8-10	3	8-10	60	60	1	2-3	GUG, CS
Henwood et al,^32^ 2008	23	22	19	19	70.4	46	24	2	3	8	3	8	45,60,75	75	ARAP	3	CS, SC
Reid et al,^33^ 2008	23	22	21	21	74.2	36	12	3	3	8	3	8	70	70	AFAP	2	None
Katula et al,^34^ 2007	15	15	12	11	75.5	43.7	12	3	3	8-10	3	8-10	70	70	AFAP	2-3	SPF
Marsh et al,^35^ 2009	15	15	11	10	75.7	30	12	3	3	8-10	3	8-10	70	70	AFAP	2-3	SPPB, SPF^c^
Nogueira et al,^36^ 2009	12	12	11	9	66.5	100	10	2	3	8-10	3	8-10	60	60	AFAP	2-3	None
Sayers et al,^37^ 2010	14	14	9	9	72.1	42	12	3	3	12-14	3	8-10	40	80	AFAP	2	None
Correa et al,^38^ 2012	13	14	13	14	67	0	6	2	3-4	8-12	3-4	8-12	NR	NR	1	2	CS
Zech et al,^39^ 2012	24	23	18	20	77.51	31.5	12	2	2	15 - 6	2	15 − 6	10-12 RPE-16	10-12 RPE-16	1	2-3	SPPB, SPF
Wallerstein et al,^40^ 2012	20	20	16	14	64.25	NA	16	2	3-4	7-4	2-4	7-4	30-50	70-90	AFAP	2	None
Pamukoff et al,^41^ 2014	10	10	8	7	70.8	55	6	3	3	8-10	3	8-10	50	50	AFAP	2-4	None
Lopes et al,^42^ 2016	20	20	12	14	68.4	0	12	3	3-4	4-6	3	8	40	60	AFAP	3	GUG, CS
Tiggemann et al,^43^ 2016	15	15	12	13	65	0	12	2	2-3	15-12-8	2-3	15-12-8	45-55-65	45-55-65	AFAP	2	GUG, SC
Richardson et al,^44^ 2018	11	11	10	10	66.5	50	10	1	3	14	3	7	40	80	AFAP	3	GUG, CS
Gray et al,^45^ 2018	34	41	20	25	81.71	31	24	2	3	10	3	10	50	80	AQAP	2	GUG, CS
Monteiro et al,^46^ 2019	20	20	20	20	66.7	0	32	3	3-4	3-6	2-3	8-12	40-60	40-60	AFAP	2	GUG, CS, ST
Jaque et al,^47^ 2020	18	17	14	12	71.61	0	12	3	3	8-12-18-25	3	8-12-18-25	BW	BW	AFAP	1-3	TUG
Müller et al,^48^ 2020^49^; 2021^49^	20	20	17	18	65.8	100	12	2	3-4	6-8	2-4	6-15	40-60	65-80	MS	2	GUG, SC, CS
Coelho-Júnior and Uchida,^50^ 2021	13	13	11	11	65	9	16	3	8	3-5	4	8-10	70-75	70-75	AFAP	2.5	TUG, CS

Abbreviations: AFAP, as fast as possible; AQAP, as quickly as possible; ARAP, as rapidly as possible; BW, body weight; CS, chair stand; GUG, 8ft Get Up & Go; Int, intervention; MS, maximum speed; NA, not applicable; NR, not reported; RPE-16; rating of perceived exertion; SC, stair climb; SPF, self-reported physical function; SPPB, Short Physical Performance Battery; ST, step test; TUG, Timed Up & Go.

^a^ Thirteen studies with physical function outcomes.

^b^ Three studies with self-reported function outcomes.

^c^ Sample size for SPPB.

#### Intervention

Most RCTs lasted 12 weeks with a frequency of 2 times per week (range, 1-3 days per week). Most trials performed 3 sets for 8 to 10 repetitions (range, 2-4 sets) with an intensity ranging from 40% to 70%, conducted in a research-type setting. Power for most of the studies involved specific instructions to move the weights “as fast or as quickly as possible” on the concentric action and 2 to 3 seconds for the eccentric action. Most trials used resistance training machines, 4 used pneumatic machines,^30,33,35,37^ 1 used free weights,^45^ 1 used body weight,^47^ and 1 used vests.^50^ The comparison group mainly used the same exercises but performed the exercises at a slower speed (2-3 seconds).

#### Outcomes

Thirteen of 20 RCTs reported physical function outcomes and 3 studies described self-reported function. Only 2 of 13 studies (15%) used a composite outcome, such as Short Physical Performance Battery Score, while the rest used single functional outcomes, mainly the 8ft Get Up & Go (GUG) test and chair stands. Secondary outcomes for strength and power mainly included lower body exercises, such as leg press and leg extension. Muscle mass was primarily assessed using dual-energy x-ray absorptiometry, gait speed was evaluated using a 6-minute walk, and balance was measured mainly using static, standing measures (eTable 2 in the Supplement).

### Primary Outcomes

**Figure 2.**
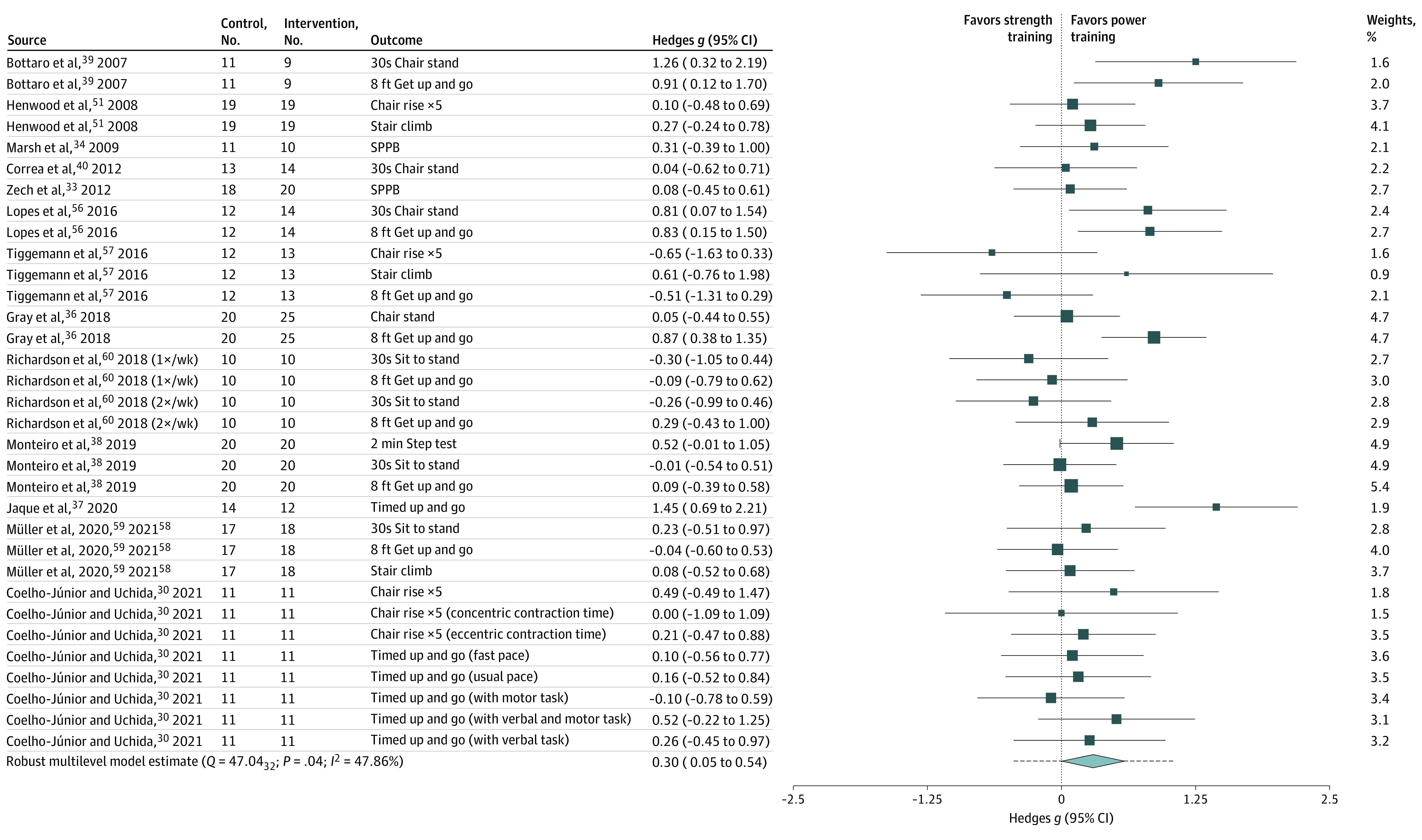
Power Training vs Traditional Strength Training Association With Physical Function Outcomes The box sizes reflect a study’s relative weight. The diamond represents the aggregate standardized mean difference and 95% CI and the dotted line represents prediction interval. SPPB indicates short physical performance battery.

**Figure 3.**
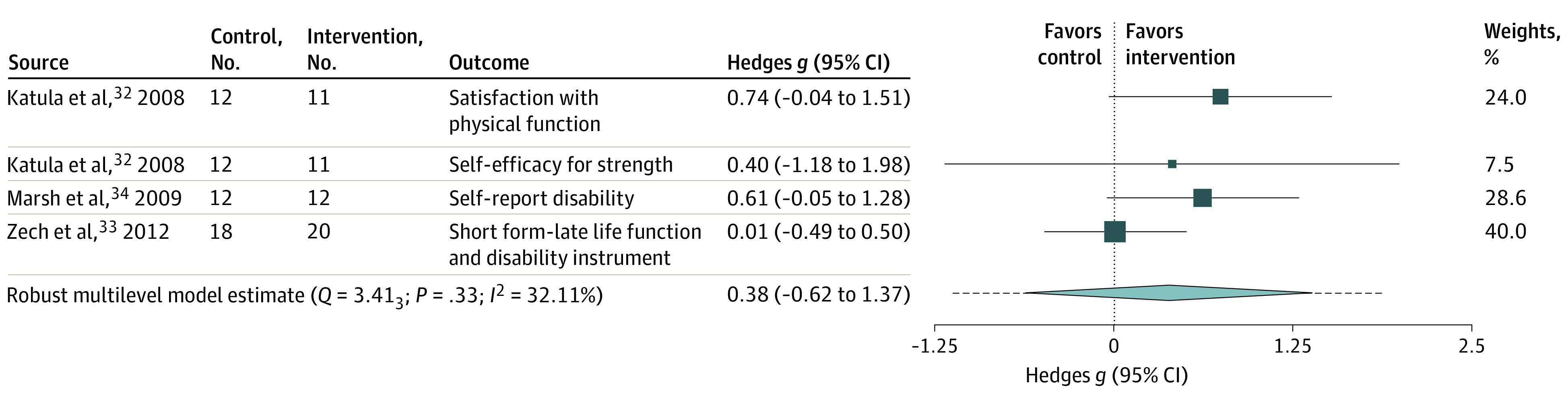
Power Training vs Traditional Strength Training Association With Self-reported Physical Function Outcomes The box size reflects study’s relative weight. The diamond represents the aggregate standardized mean difference and 95% CI and the dotted line represents prediction interval.

The main model for physical function (33 outcomes across 13 RCTs with 383 participants [median, 2; range, 1-8 outcomes per study]) revealed a small SMD (0.30; 95% CI, 0.05-0.54), with moderate heterogeneity (*Q* = 47.04; *P* = .04; *I*^2^ = 48; low certainty) (Figure 2). The main model for self-reported physical function (4 outcomes across 3 studies including 85 participants: median, 1; range, 1-2 outcomes per study) revealed a small SMD favoring PT (SMD, 0.38; 95% CI, –0.62 to 1.37), with moderate heterogeneity (*Q* = 3.41, *P* = .33; *I*^2^ = 32%; low certainty) (Figure 3).

### Secondary Outcomes

For power, the main model for all pooled power outcomes (76 across 15 studies: median, 2; range, 1-12 outcomes per study) revealed a moderate association favoring PT (SMD, 0.44; 95% CI, 0.21-0.66; *I*^2^ = 47%; low certainty). For strength (87 across 15 studies: median, 2; range, 1-20 outcomes per study) showed no association (SMD, 0.01; 95% CI, –0.14 to 0.16; *I*^2^ = 25%; low certainty). Muscle mass and size (21 across 10 studies: median, 1; range, 1-8 outcomes per study) showed no association. (SMD, 0.0004; 95% CI, –0.08 to 0.08; *I*^2^ = 0%; low certainty). Gait speed (16 across 6 studies: median, 1.5; range, 1-8 outcomes per study) showed no association (SMD, –0.03; 95% CI, –0.16 to 0.10; *I*^2^ = 17%; low certainty). Balance (14 across 5 studies: median, 2; range, 1-5 outcomes per study) showed no association (0.05; 95% CI, –0.82 to 0.92; *I*^2^ = 74%; very low certainty).

#### Adverse Events

Adverse events were insufficiently reported (14 of 20 [70%] RCTs). Adverse events per 1000 person-sessions were relatively low and showed minimal difference between conditions, with values of 3.27 (95% CI, 1.76-6.09) for PT and 2.08 (95% CI, 0.99-4.36) for traditional training. Events mainly included exacerbation of arthritis and knee or muscle soreness. Studies with low functioning participants reported more adverse events. There were no serious adverse events reported for either group. Adherence to PT was 82.5% (95% CI, 74.2%-88.6%) and for traditional training was 81.8% (95% CI, 70.1%-89.4%).

### Moderators

Subgroup and meta-regression models (eTable 3 in the Supplement) for preregistered physical function outcomes showed only weekly frequency of training appeared to have a significant association, with greater frequencies of training showing a greater benefit for PT (β = 0.36; –0.009 to 0.73; *P* = .05).

### RoB 2 and GRADE Assessment

RoB 2 indicated high risk for 5 studies, some concerns for 6 studies, and low risk for 2 studies for the physical function outcome (eTable 4 and justifications in eAppendix 2 in the Supplement). Overall, we judged the physical function outcome as high risk of bias. Self-reported function was judged to be at some concern. We judged the measurement outcome domain to be low risk for self-reported outcome since both groups were exercising and assessors were blinded. Although not reported in the articles, 5 RCTs confirmed (via our email communication with the authors) that assessors were blinded.^31,35,38,46^ If raw unadjusted scores of registered outcomes were reported, we rated the selective reporting domain as low risk. We did not analyze the risk of bias due to selective nonreporting or underreporting because that type of bias is not covered in RoB 2.

Using GRADE (eTable 5 in the Supplement), we judged the certainty in our estimates to be low across primary outcomes. For physical function, we downgraded the evidence by 1 level for serious risk of bias and 1 level for serious imprecision owing to the low numbers of participants (<800). For self-reported function, we downgraded the evidence by 2 levels owing to very serious imprecision and was judged as low certainty.

The contour-enhanced funnel plot for both preregistered outcomes and all outcomes is shown in eFigure 2 in the Supplement. Inspection of the funnel plot did not reveal any obvious small-study bias.

### Sensitivity Analyses

Subgroup analysis showed that the effect size estimate was lower for RCTs coded as only low risk or some concerns (0.18; 95% CI, –0.06 to 0.42) compared with those with high risk (0.48; 95% CI, –0.16 to 1.12), but the comparison was relatively imprecise (high risk minus low or some risk: –0.30; 95% CI, –0.80 to 0.19). Individual test of function showed similar results for GUG and stair climb, but the effect size for chair stand was smaller and imprecise. Dropping the influential study and using *r* = 0.5 as pre-post correlation did not change the inference for the primary outcomes (eTable 6 in the Supplement).

### Protocol and Review Differences

For physical function, we included composite outcomes (eg, Short Physical Performance Battery Score) preferentially if reported, and if they were not reported, we included all other power-based outcomes (chair rise, up and go tests, stair climb). Moderators were operationalized as categorical variables; however, we opted to analyze some variables continuously when deemed appropriate. We also included GRADE to assess the certainty of the evidence.

## Discussion

Our systematic review and meta-analysis assessed the data for PT and physical function. Based on low-certainty evidence, our findings suggest that PT was associated with an improvement in physical function and self-reported function to a greater extent than traditional strength training.

Previous systematic reviews and meta-analyses reported similar effect sizes of SMD (0.41; 95% CI, 0.18-0.65)^15^ and 0.32 (95% CI, 0.06-0.65)^16^ for PT. However, our evaluation was restricted to studies solely using PT and excluded studies specifically using plyometric exercises or dissimilar exercises in the comparison group. We excluded 2 studies included in previous reviews that performed functional, standing exercises in the PT group, but the control group performed seated exercises.^20,52^ We also removed 2 studies that performed counter movement jumps in the PT group^17,18^ and another study that combined circuit training with PT.^53^ Thus, our review attempted to isolate the true outcome of PT from multi-interventional training.

To assess the risk of bias, we used the revised Cochrane RoB 2.^23^ Previous systematic reviews and meta-analyses either used no formal RoB 2 assessment or used the PEDro scale, which combines both reporting and methodological limitations into a single scale. Unlike previous reviews, we evaluated the certainty (or confidence) in the body of evidence using the GRADE method.^24^ We also included self-reported function as a primary outcome. Unlike performance measures, self-reported measures assess an individual’s function in their lived or actual environment and are increasingly recognized by government regulatory agencies to comprehensively assess function.^54^ Thus, our study reports the outcome of PT per se on both objective and subjective function, along with the use of rigorous tools for assessing RoB 2 and the certainty of evidence.

Most of the studies used the GUG test and chair stands to assess functional outcomes. Chair stands and GUG are performed at a fast pace; the Timed Up & Go test is performed at a usual pace. To assess the clinical relevance of these measures, we back-transformed the SMD to natural units for the most common tests: based on SDs from a large, observational cohort of community-living older adults,^55^ for GUG (SMD, 0.34), we estimated a mean (SD) 0.62 (1.85)-second improvement, and for chair stands (SMD, 0.13), a 0.56 (4.3)-stands improvement.

For secondary outcomes, PT showed an SMD of 0.44 (95% CI, 0.21-0.66) in lower body power, which is consistent with a previous systematic review and meta-analysis (SMD, 0.42; 95% CI, –0.02 to 0.85).^15^ Power training has been shown to increase the cross-sectional area and power of type 2 muscle fibers, independent of sex or age.^56^ Furthermore, there is a preferential loss of type 2 fibers with aging.^57^ The multicenter LIFE study showed that improvements in muscle strength and power, as seen in the improvements in chair stands, is largely responsible for the reduction of mobility disability in the physical activity group.^58^ Thus, an increase in muscle power offers a biological basis for the improvement observed in function.

For other secondary outcomes, data suggest that gait speed, strength, and muscle mass were not associated. The intensity and volume used was similar for both groups for most studies; hence, similar findings in strength and muscle mass may be expected. Most studies showing improvements in habitual gait speed used multicomponent exercise programs, such as over-ground walking.^59,60^ The results of secondary outcomes are consistent with a previous meta-analysis.^15^ Adverse events were minimal and similar between the groups.

### Limitations

This study has limitations. We did not include RCT registries or ongoing studies and limited our search to English-language publications. Both RoB 2 and GRADE assessments require judgment (ie, subjective) and could differ across people. We used multiple scales to assess the construct of physical function, using SMD as recommended; however, the interpretability is diminished although we back-transformed to original scales. Some of the study-level limitations include (1) participants were instructed to move the weights as fast as possible, but none of the studies objectively tracked velocity to corroborate the increase in velocity during training; (2) most trials were short (12 weeks) and had small sample sizes; and, (3) although resistance training is considered safe,^61^ adverse effects were insufficiently documented and reported across trials. Overall, there was insufficient information in the included RCTs to judge randomization and allocation concealment domain, blinded assessors’ domain, preregistration, and adverse events.

## Conclusions

We recommend that future PT studies obtain larger and better-justified sample sizes, measure both performance and self-reported functional outcomes, track power during workout sessions using an objective measure, and emphasize the proper conduct and reporting of important methodological domains. Power training requires the person to move the weight faster in the lifting phase. This directive can be challenging, especially in individuals who are very old or cognitively impaired. Nevertheless, like traditional strength training, PT can be performed using weight machines, pneumatic machines, or body weight, and thus there are no added cost or feasibility issues. Furthermore, there was no increase in adverse effects or serious adverse events reported. However, the low number of adverse events should be interpreted cautiously owing to the small sample size of the included studies and insufficient reporting. It would be prudent for benefits, harms, cost, and client values/preferences to be evaluated before PT practice guidelines provide recommendations on PT in older adults.

Based on the available evidence, our systematic review and meta-analysis suggests that PT was associated with a modest improvement in physical function compared with traditional strength training in healthy older adult participants in RCTs. However, robust, large trials are warranted to make definitive statements.

## Notes

Supplement. **eTable 1.** Reasons for Exclusions **eFigure 1.** Flow Diagram of Trial Identification and Selection for the Updated Search **eTable 2.** Primary and Secondary Outcomes in Studies **eTable 3.** Sub-group and Meta-Regression Analyses **eTable 4.** RoB for Primary Outcomes **eTable 5.** GRADE Summary of Findings **eTable 6.** Sensitivity Analysis **eFigure 2.** Funnel Plot for All Effects **eAppendix 1.** Search Strategy **eAppendix 2.** RoB Analyses
