# Supplementary material for: Comparison of Power Training vs Traditional Strength Training on Physical Function in Older Adults: A Systematic Review and Meta-analysis
Source: JAMA Netw Open. 2022 May 11;5(5):e2211623. doi: 10.1001/jamanetworkopen.2022.11623 (PMC9096601; doi:10.1001/jamanetworkopen.2022.11623)
Supplement: Supplement. — eTable 1. Reasons for Exclusions eFigure 1. Flow Diagram of Trial Identification and Selection for the Updated Search eTable 2. Primary and Secondary Outcomes in Studies eTable 3. Sub-group and Meta-Regression Analyses eTable 4. RoB for Primary Outcomes eTable 5. GRADE Summary of Findings eTable 6. Sensitivity Analysis eFigure 2. Funnel Plot for All Effects eAppendix 1. Search Strategy eAppendix 2. RoB Analyses [file jamanetwopen-e2211623-s001.pdf]

Supplemental Online Content

Balachandran AT, Steele J, Angielczyk D, et al. Comparison of power training vs traditional strength training on physical function in older adults: a systematic review and meta-analysis. *JAMA Netw Open*. 2022;5(5):e2211623. doi:10.1001/jamanetworkopen.2022.11623

eTable 1. Reasons for Exclusions
eFigure 1. Flow Diagram of Trial Identification and Selection for the Updated Search
eTable 2. Primary and Secondary Outcomes in Studies
eTable 3. Sub-group and Meta-Regression Analyses
eTable 4. ROB for Primary Outcomes
eTable 5. GRADE Summary of Findings
eTable 6. Sensitivity Analysis
eFigure 2. Funnel Plot for All Effects
eAppendix 1. Search Strategy
eAppendix 2. ROB Analyses

This supplemental material has been provided by the authors to give readers additional information about their work.

© 2022 Balachandran AT et al. *JAMA Network Open*.

eTable 1. Reasons for exclusions

1.	Miszko_2003[1]	Intervention	Used jump squats
2.	Balachandran_2014[2]	Intervention	Used circuit training in addition to power training in the power group using pneumatics. Control group used weight machines and had no circuit training.
3.	Bean_2009[3]	Intervention	Exercises different between groups. exercises were based on normal functional task. Control grp performed seated exercises
4.	Bean_2004[4]	Intervention	Exercises different between groups. exercises were based on normal functional task. Control grp performed seated exercises
5.	Ramirez-Campillo 2014[5]	Intervention	Power training involved counter movement jumps
6.	Yoon_2017[6]	Population	Population with mild cognitive impairement
7.	Englund_2017[7]	Intervention	Isokinetic exercises with same instructions for both groups
8.	Macaluso_2003[8]	Intervention	same speed for both groups. During each set, all participants were required to pedal as fast as possible
9.	Richardson_2018[9]	Design	Cross over trial
10.	Hart_2003[10]		Journal club abstract
11.	Vilada_2007[11]	Design	Not randomized.
12.	Drey_2012[12]	Duplicate	Used Zech_2012 instead since same data
13.	Vieira_2021[13]	Design	Not randomized

1. Miszko TA, Cress ME, Slade JM, Covey CJ, Agrawal SK, Doerr CE. Effect of strength and power training on physical function in community-dwelling older adults. *J Gerontol A Biol Sci Med Sci*. 2003;58(2):171-175.

2. Balachandran A, Krawczyk SN, Potiaumpai M, Signorile JF. High-speed circuit training vs hypertrophy training to improve physical function in sarcopenic obese adults: A randomized controlled trial. *Exp Gerontol*. 2014;60:64-71.

3. Bean JF, Kiely DK, LaRose S, O'Neill E, Goldstein R, Frontera WR. Increased velocity exercise specific to task training versus the national institute on aging's strength training program: Changes in limb power and mobility. *J Gerontol A Biol Sci Med Sci*. 2009;64(9):983-991.

4. Bean JF, Herman S, Kiely DK, et al. Increased velocity exercise specific to task (InVEST) training: A pilot study exploring effects on leg power, balance, and mobility in community-dwelling older women. *J Am Geriatr Soc*. 2004;52(5):799-804.

5. Ramirez-Campillo R, Castillo A, de la Fuente CI, et al. High-speed resistance training is more effective than low-speed resistance training to increase functional capacity and muscle performance in older women. *Exp Gerontol*. 2014;58C:51-57.

6. Yoon DH, Kang D, Kim H, Kim J, Song HS, Song W. Effect of elastic band-based high-speed power training on cognitive function, physical performance and muscle strength in older women with mild cognitive impairment. *Geriatrics & gerontology international*. 2017;17(5):765-772.

© 2022 Balachandran AT et al. *JAMA Network Open*.

7. Englund DA, Sharp RL, Selsby JT, Ganesan SS, Franke WD. Resistance training performed at distinct angular velocities elicits velocity-specific alterations in muscle strength and mobility status in older adults. *Exp Gerontol*. 2017;91:51-56.

8. Macaluso A, Young A, Gibb KS, Rowe DA, De Vito G. Cycling as a novel approach to resistance training increases muscle strength, power, and selected functional abilities in healthy older women. *J Appl Physiol (1985)*. 2003;95(6):2544-2553.

9. Richardson DL, Duncan MJ, Jimenez A, Jones VM, Juris PM, Clarke ND. Movement velocity during high-and low-velocity resistance exercise protocols in older adults. *Exp Gerontol*. 2018;107:140-147.

10. Hart LE. High-velocity resistance training for increasing peak muscle power in elderly women. *Clinical Journal of Sport Medicine*. 2003;13(1):66.

11. Villada J, Da Silva M, Alonso J. Influence of a training programme with jumps on explosive force, speed of movement and dynamic balance in the elderly. *REVISTA ESPANOLA DE GERIATRIA Y GERONTOLOGIA*. 2007;42(4):218.

12. Drey M, Zech A, Freiberger E, et al. Effects of strength training versus power training on physical performance in prefrail community-dwelling older adults. *Gerontology*. 2012;58(3):197-204.

13. Vieira IP, Lobo PC, Fisher J, Ramirez-Campilo R, Pimentel GD, Gentil P. Effects of high-speed versus traditional resistance training in older adults. *Sports Health*. 2021:19417381211015211.

© 2022 Balachandran AT et al. *JAMA Network Open*.

eFigure 1. Flow Diagram of Trial Identification and Selection for the updated search (October 2019 – October 2021)

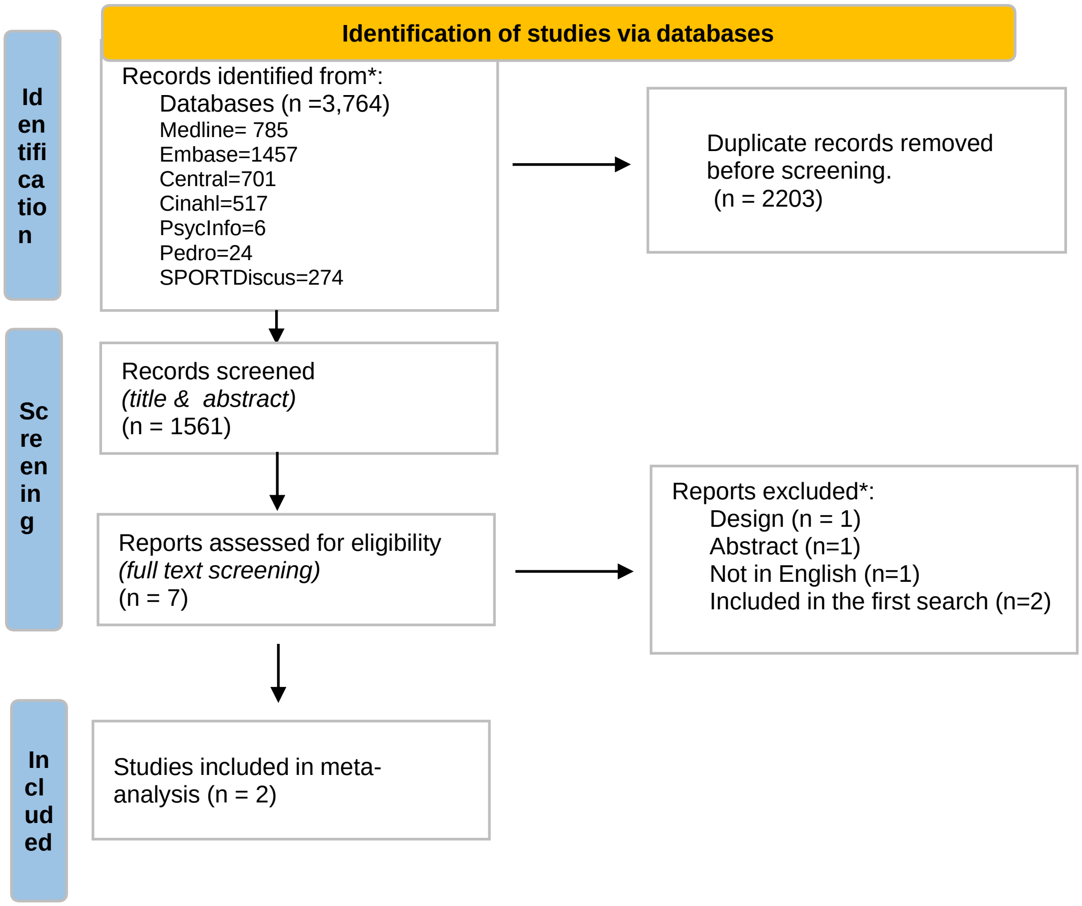

© 2022 Balachandran AT et al. *JAMA Network Open*.

eTable 2. Primary and secondary outcomes in studies

Study	Physical function	Power	Strength	Gait Speed	Muscle	Balance
Feilding_2003	None	LP, KE	LP,KE	-	-	-
Bottaro_2007	GUG,CS	LP,CP	LP,CP	-	--	-
Henwood_2008	CS,SC	CP,LP,BC,LE,LC	BP,R,BC,LP,LC,LE	400m, 6m	LBM (DXA)-	FR
Reid_2008	None	KE,LP	LP,KE	-	-	-
Katula_2008	SPF	--	-	-	-	-
Marsh_2009	SPPB, SPF	LP,KE	LP,KE	-	LBM (DXA)	-
Nogueira_2009	None	LP,CP	LP,CP		RFT,BBT (US)	
Sayers_2010	None	KE		-	-	-
Correa_2012	CS	CMJ	KE		VLT,VMT,RFT (US)	-
Zech_2012	SPPB, SPF	STS	-	4m	ALM (DXA)	SB
Wallerstein_2012	None	-	LP,CP		QCSA (MRI)	-
Pamukoff_2014	None	LP, LE	LE,LP			FL,LL
Lopez_2016	GUG,CS	KE,KF,HF,HE,DF	KE,KF,LP	6min	-	SI,PP,SP,TT
Tiggerman_2016	GUG,SC	SJ,CMJ	LP,KE	6min	-	-
Richardson_2018	GUG,CS	-	LP,LE,CR,GS, R,CP,AC, TE	6min	FFM (BIA)	
Gray_2018	GUG,CS	-	CP,KC,BO, LR,TE	-	LBM (DXA)	-
Monteiro_2019	GUG,CS,ST	-	-	-	LBM (DXA)	-
Jaque_2020	TUG	STS	-	-	-	-
Coelho-Júnior_2021	TUG,CS	STS	GS,KE,HF, AE	6 min	-	OLS,SB

© 2022 Balachandran AT et al. *JAMA Network Open*.

Müller_2020	GUG, SC, CS	KE,CMJ,CE	LP,KE	-	LBM (DXA), RFT, VLT,VMT	-
Total studies	13,3	15	15	6	10	5

NOTE: There could be more than one measure for the above measures in one study. For ex, LP power at 70% and 40% 1RM, 6m usual and fast-paced walk

Strength & Power - LP: Leg press, LE: Leg extension, LC: Leg curls, KE: Kee Extension, KF: Knee flexion, KC: Knee curls, HF: Hip flexion, HE: Hip extension, DF: Dorsi flexion, AE: Ankle extension, STS: Sit to Stand, CMJ: Counter movement jump, SJ: Standing jump, CE: Cycle ergometer.
CP: Chest press, BC: Bicep's curl, BP: Bench press, GS: Grip strength, R: Rows, AC: Arm curls, BO: Bent over Row, LR: Lateral raise, TE: Triceps extension

Muscle mass - LBM: Lean body mass, FFM: Fat free mass, ALM: Appendicular lean mass , VLT: Vastus Lateralis thickness, VLM: Vastus medialis thickness, RFT: Rectus femoris thickness, BBT: Biceps brachii thickness, QCSA: Quadriceps cross sectional area , DXA: Dual-energy x-ray absorptiometry , US: Ultrasound, BIA: Bioelectrical Impedance Analyzer, MRI: Magnetic Resonance Imaging

Balance – FR: Functional reach, SB: Standing balance, FL: Forward lean, LL: Lateral lean, OLS: One legged stance, SI: Step Initiation, PP: Preparation phase, SP: Swing phase, TT: Total time.

© 2022 Balachandran AT et al. *JAMA Network Open*.

eTable 3. Sub-group and meta-regression analyses[a]

**Subgroups**	**Estimate (95% CI)**
High vs. Low Risk of Bias (ROB)	0.48 (-0.16, 1.12) High ROB<br>0.18 (-0.06, 0.42) Low ROB<br>-0.30 (-0.80, 0.19), P = 0.23 (contrasts)
High vs Low function	0.34 (-0.004, 0.69) High function<br>0.19 (0.067, 0.31) Low function<br>-0.15 (-0.46, 0.15), P = 0.32 (contrasts)
**Outcomes**[b]	**Coefficients (95% CI)**
Age	0.02 (-0.01, 0.05), P = 0.26
BMI	-0.06 (-0.20, 0.07), P = 0.31
Sex	0.002 (-0.008, 0.013), P = 0.65
Duration	0.001 (-0.01, 0.01), P = 0.88
Frequency	0.36 (-0.009, 0.73), P = 0.05
Relative Load	0.005 (-0.01, 0.02), P = 0.49

NOTE: a. Full output available at https://osf.io/syjnx/

b. Meta-regression scatter plots available in OSF under "Secondary Analyses" folder: https://osf.io/uzqxj/.

© 2022 Balachandran AT et al. *JAMA Network Open*.

eTable 4. ROB for primary outcomes

1. ROB for physical function_outcome

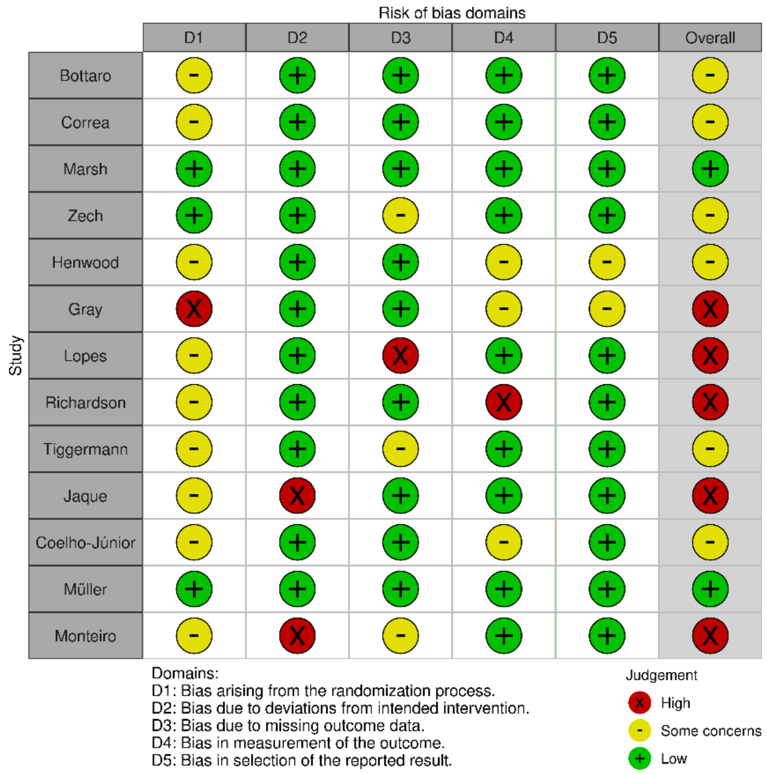

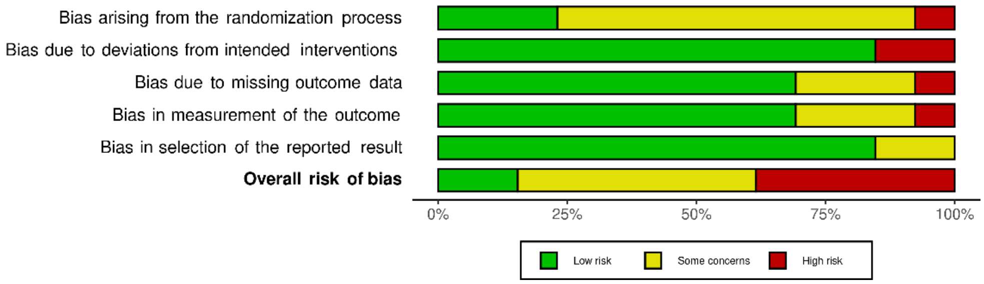

© 2022 Balachandran AT et al. *JAMA Network Open*.

2. ROB for self-reported physical function outcome

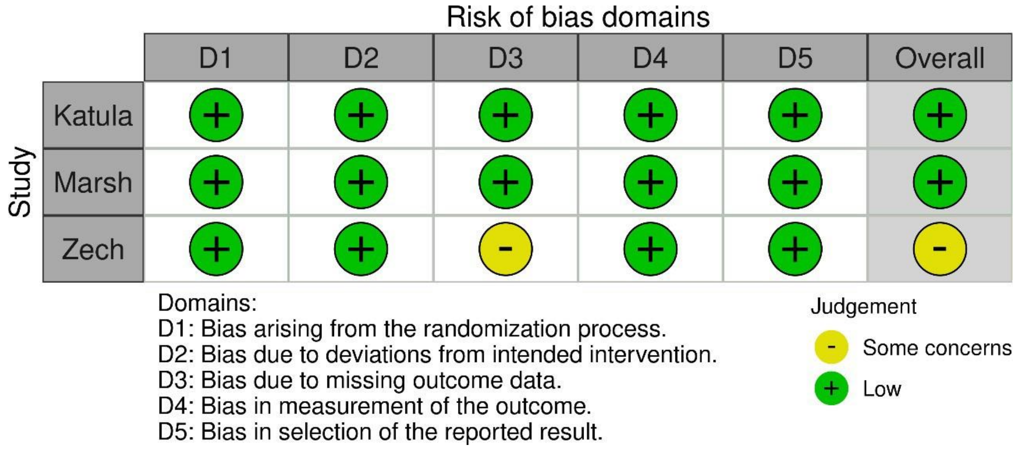

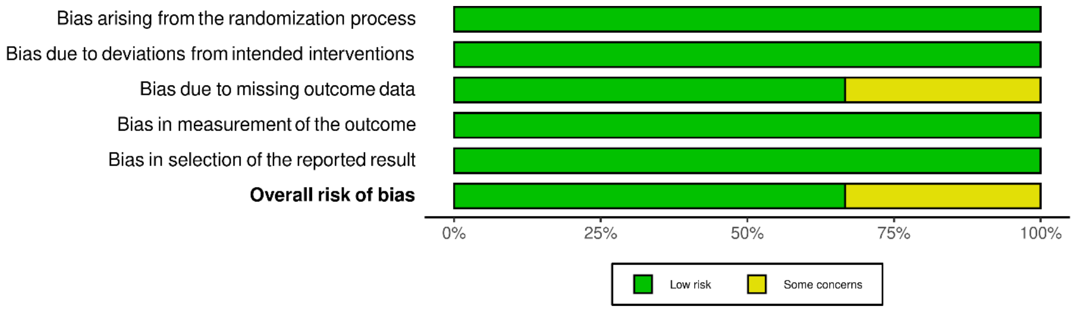

© 2022 Balachandran AT et al. *JAMA Network Open*.

Secondary Outcomes

Power

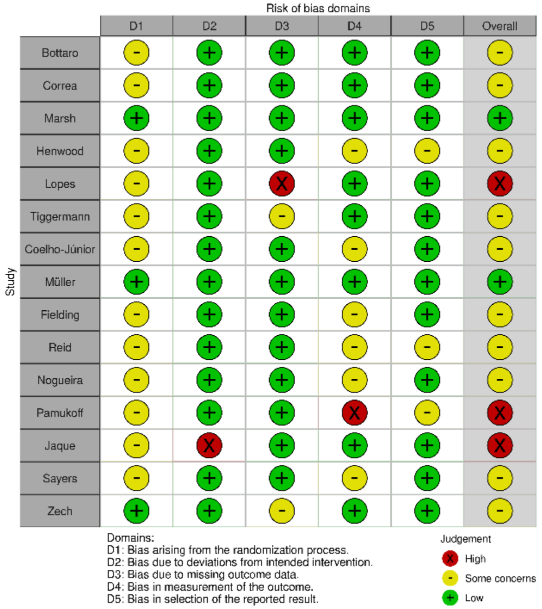

Strength

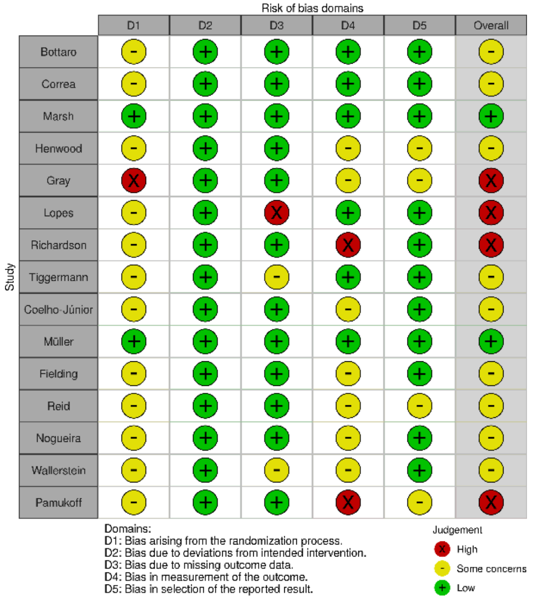

Muscle

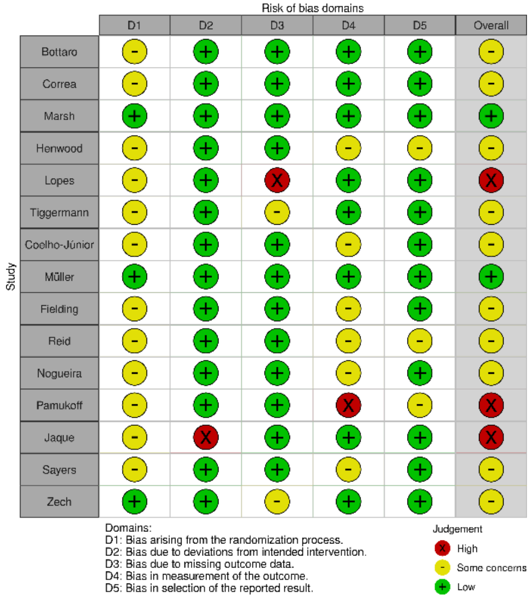

Gait

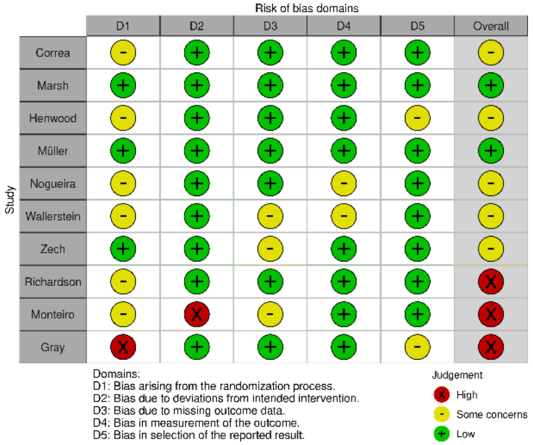

Balance

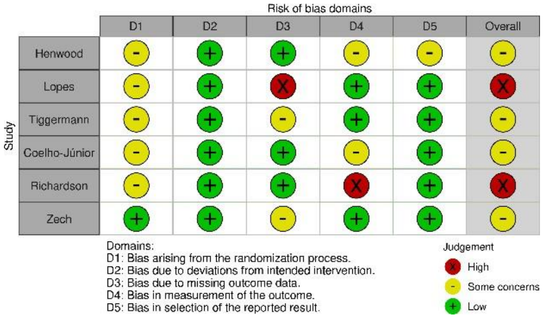

© 2022 Balachandran AT et al. *JAMA Network Open*.

eTable 5. GRADE summary of findings

Certainty Assessment								
Outcomes	Participants (studies)	Risk of bias	Inconsistency	Indirectness	Imprecision	Publication bias	Overall certainty	Absolute effects 95%CI
Physical Function	403 (13 RCTs)	serious[a]	not serious I^2 = 48%	not serious	serious[b]	undetected	⊕⊕◯◯ Low	SMD **0.30 SD higher** (0.05 higher to 0.54 higher)
Self-reported function	85 (3 RCTs)	not serious	not serious I^2 = 32%	not serious	very serious[bc]	undetected	⊕⊕◯◯ Low	SMD **0.38 SD higher** (0.62 lower to 1.37 higher)
**Secondary outcomes**								
Power	409 (15 RCTs)	serious[a]	not serious I^2 = 47%	not serious	serious[b]	undetected	⊕⊕◯◯ Low	SMD **0.44 SD higher** (0.21 higher to 0.66 higher)
Strength	433 (15 RCTs)	serious[a]	not serious I^2 = 25%	not serious	serious[b]	undetected	⊕⊕◯◯ Low	SMD **0.01 SD lower** (0.14 lower to 0.16 higher)
Muscle	336 (10 RCTs)	serious[a]	not serious I^2 = 0%	not serious	serious[b]	undetected	⊕⊕◯◯ Low	SMD **0.0004 SD lower** (0.08 lower to 0.08 higher)
Gait speed	189 (6 RCTs)	serious[a]	not serious I^2 = 17%	not serious	serious[b]	undetected	⊕⊕◯◯ Low	SMD **0.03 SD lower** (0.16 lower to 0.10 higher)
Balance	139 (4 RCTs)	serious[a]	serious[d] I^2 = 74%	not serious	very serious[bc]	undetected	⊕◯◯◯ Very low	SMD **0.05 SD higher** (0.82 lower to 0.92 higher)

CI: confidence interval; **SMD:** standardized mean difference

Explanations

a. Downgraded by one level for serious risk of bias

b. Downgraded by one level because Optimum Information Size (OIS) less than 800 participants

c. Downgraded by 2 levels due to CI including appreciable harm and appreciable benefit

d. Downgraded for high inconsistency (I^2 = 74%)

NOTE: For imprecision, we used the null effect threshold for primary outcomes and a small effect threshold (0.20) for secondary outcomes. We used the optimum information size (OIS) of <800 participants for rating down as recommended. For risk of bias, we downgraded when most studies had high ROB or some concerns. For the rest and overall certainty, we followed the GRADE recommendations.

© 2022 Balachandran AT et al. *JAMA Network Open*.

eTable 6. Sensitivity analysis

Single function tests[a]

Outcomes	Estimate (95% CI)
Get up & go	0.34 (0.04, 0.63), I^2= 54%
Chair stands	0.13 (-0.06, 0.32), I^2 = 0%
Stair climb	0.32 (0.11, 0.52), I^2 = 28%

a. Full output available at https://osf.io/sutzf/

Dropping Influential study for primary outcomes[bc]

Outcomes	Estimate (95% CI)
Physical function	0.23 (0.03, 0.43), I^2 = 31%
Self-reported physical function	0.64 (0.27, 1.0), I^2 = 32%

b. Full output available at https://osf.io/sutzf/
c. Hat values & Cook's distances at https://osf.io/ndqwb/

Pre-post correlation using r = 0.5[c]

Outcomes	Estimate (95% CI)
Physical function	0.28 (0.06, 0.49), I^2 = 22%
Self-reported physical function	0.36 (-0.64, 1.36), I^2 = 9%

c. Full output available at https://osf.io/jqhn2/

Pre-post correlation using r = 0.9[d]

Outcomes	Estimate (95% CI)
Physical function	0.31 (0.05, 0.56), I^2 = 76%
Self-reported physical function	0.40 (-0.60, 1.39), I^2 = 64%

d. Full output available at https://osf.io/brkax/

© 2022 Balachandran AT et al. *JAMA Network Open*.

eFigure 2. Funnel plot

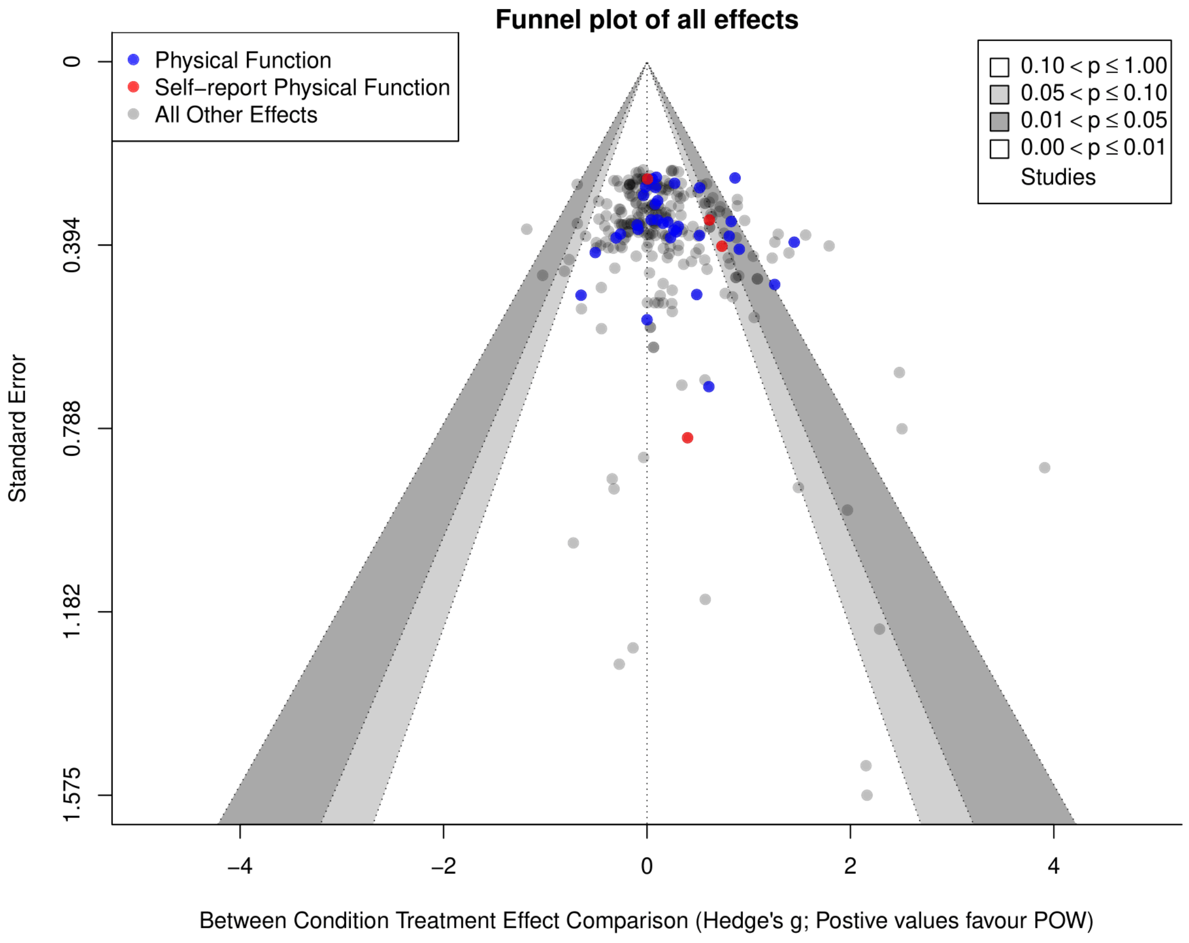

© 2022 Balachandran AT et al. *JAMA Network Open*.

eAppendix 1. Search strategy

Medline

middle aged/ or exp aged/ or exp geriatrics/ or healthy aging/ or exp aging/	5016755
independent living/ or "housing for the elderly"/	6252
(middleage* or middle age* or old age or midlife or aged or aging or ageing or elderly or elders or senior or seniors or geriatric* or older or late life or lat	1231590
((community or independent or solo or alone) adj3 (dwelling or living)).ti,ab,kf	33161
or/1-4	5557844
resistance training/	7685
((power or high-velocity or velocity or ballistic or explosive*) adj5 (train* or lift* or resistance or concentric or exerci*)).ti,ab,kf	8104
(high-speed resistance).ti,ab,kf	19
((fast or quick or speed* or velocity) adj2 (reps or repetition*)).ti,ab,kf	220
(complex training or contrast training or speed-strength).ti,ab,kf	318
or/6-10	15519
(controlled clinical trial or randomized controlled trial).pt.	577916
clinical trials as topic.sh.	188422
(randomi#ed or randomly or RCT$1 or placebo*).tw.	922278
((singl* or doubl* or trebl* or tripl*) adj (mask* or blind* or dumm*)).tw.	166220
trial.ti.	204605
or/12-16	1294874
5 and 11 and 17	2322
exp Animals/ not (exp Animals/ and Humans/)	4616467
(exp child/ or exp infant/ or exp adolescent/) not ((exp child/ or exp infant/ or exp adolescent/) and (exp aged/ or exp adult/))	1821295
(comment or editorial or interview or news).pt.	1359599
(letter not (letter and randomized controlled trial)).pt.	1037208
18 not (19 or 20 or 21 or 22)	2251

© 2022 Balachandran AT et al. *JAMA Network Open*.

Embase

middle aged/ or exp aged/ or exp geriatrics/ or exp aging/	3814485
independent living/ or "home for the aged"/	14403
(middleage* or middle age* or old age or midlife or aged or aging or ageing or elderly or elders or senior or seniors or geriatric* or older or late life or lat	1659624
((community or independent or solo or alone) adj3 (dwelling or living)).ti,ab,kw	42996
or/1-4	4674338
resistance training/	16235
((power or high-velocity or velocity or ballistic or explosive*) adj5 (train* or lift* or resistance or concentric or exerci*)).ti,ab,kw	9740
(high-speed resistance).ti,ab,kw	19
((fast or quick or speed* or velocity) adj2 (reps or repetition*)).ti,ab,kw	241
(complex training or contrast training or speed-strength).ti,ab,kw	365
or/6-10	25555
exp controlled clinical trial/	756643
exp "clinical trial (topic)"/	307229
(randomi#ed or randomly or RCT$1 or placebo*).tw.	1299701
((singl* or doubl* or trebl* or tripl*) adj (mask* or blind* or dumm*)).tw.	230358
trial.ti.	281254
or/12-16	1845103
5 and 11 and 17	3124
(exp Animal/ or nonhuman/) not ((exp Animal/ or nonhuman/) and exp Human/)	6207141
(exp child/ or exp adolescent/) not ((exp child/ or exp adolescent/) and (exp adult/))	2051653
(editorial or note).pt.	1404358
letter.pt not (letter.pt and randomized controlled trial/)	1081642
18 not (19 or 20 or 21 or 22)	2994

Central

[mh ^"middle aged"] or [mh aged] or [mh geriatrics] or [mh ^"healthy aging"] or [mh aging]	1118
[mh ^"independent living"] or [mh ^"housing for the elderly"]	377
(middleage* or (middle NEAR/1 age*) or "old age" or midlife or aged or aging or ageing or elderly or elders or senior or seniors or geriatric* or older or "l	520309
((community or independent or solo or alone) NEAR/3 (dwelling or living)):ti,ab,kw	5171
{OR #1-#4}	521108
[mh ^"resistance training"]	2952
((power or high-velocity or velocity or ballistic or explosive*) NEAR/5 (train* or lift* or resistance or concentric or exerci*)):ti,ab,kw	2322
("high-speed resistance"):ti,ab,kw	16
((fast or quick or speed* or velocity) NEAR/2 (reps or repetitions)):ti,ab,kw	17
("complex training" or "contrast training" or "speed-strength"):ti,ab,kw	77
{OR #6-#10}	5041
#5 and #11	2372
([mh child] or [mh infant] or [mh adolescent]) not (([mh child] or [mh infant] or [mh adolescent]) and ([mh aged] or [mh adult]))	112976
#12 not #13	2192

© 2022 Balachandran AT et al. *JAMA Network Open*.

Cinahl

(MH "middle age") or (MH aged+) or (MH geriatrics) or (MH "healthy aging") or (MH aging+)	1152983
(MH "community living+") or (MH "housing for the elderly")	20096
(middleage* or (middle N1 age*) or "old age" or midlife or aged or aging or ageing or elderly or elders or senior or seniors or geriatric* or older or "late life" or "later life")	1320074
((community or independent or solo or alone) N3 (dwelling or living))	47778
S1 OR S2 OR S3 OR S4	1337723
(MH "resistance training")	4189
((power or high-velocity or velocity or ballistic or explosive*) N5 (train* or lift* or resistance or concentric or exerci*))	22586
("high-speed resistance")	9
((fast or quick or speed* or velocity) N2 (reps or repetition*))	112
("complex training" or "contrast training" or "speed-strength")	141
S6 OR S7 OR S8 OR S9 OR S10	26159
(PT "Clinical trial") or (PT "randomized controlled trial")	171806
(MH "Clinical Trials+")	266670
(randomised or randomized or randomly or RCT or RCTs or placebo*)	298288
((singl* or doubl* or trebl* or tripl*) N1 (mask* or blind* or dumm*))	67531
(TI trial)	94733
S12 OR S13 OR S14 OR S15 OR S16	425345
S5 and S11 and S17	2969
(MH vertebrates+) not ((MH vertebrates+) and (MH human))	167721
((MH child+) or (MH adolescence+)) not (((MH child+) or (MH adolescence+)) and (MH adult+))	525911
PT book review or commentary or editorial or interview	396408
(PT letter) not ((PT letter) and (PT "randomized controlled trial"))	273476
S18 not (S19 or S20 or S21 or S22)	2731

© 2022 Balachandran AT et al. *JAMA Network Open*.

Psycinfo

exp geriatrics/ or exp aging/ or ("360" or "380" or "390").ag	554824
(middleage* or middle age* or old age or midlife or aged or aging or ageing or elderly or elders or senior or seniors or geriatric* or older or late life or later life).ti,ab,id	463817
((community or independent or solo or alone) adj3 (dwelling or living)).ti,ab,id	17966
or/1-3	812139
((power or high-velocity or velocity or ballistic or explosive*) adj5 (train* or lift* or resistance or concentric or exerci*)).ti,ab,id	2401
(high-speed resistance).ti,ab,id	4
((fast or quick or speed* or velocity) adj2 (reps or repetition*)).ti,ab,id	78
(complex training or contrast training or speed-strength).ti,ab,id	114
or/5-8	2591
exp clinical trials/	11608
(randomi#ed or randomly or RCT$1 or placebo*).tw.	162030
((singl* or doubl* or trebl* or tripl*) adj (mask* or blind* or dumm*)).tw.	25383
trial.ti.	28591
or/10-13	176381
4 and 9 and 14	52
("20").po not ("20" and "10").po	353993
("100" or "200").ag not (("100" or "200") and "300").ag	492563
("2600" or "2800" or "3000" or "3500" or "4600" or "4800" or "5000").dt	295074
15 not (16 or 17 or 18)	50

© 2022 Balachandran AT et al. *JAMA Network Open*.

Sportdiscuss

DE "OLDER people" OR DE "EXERCISE for older people" OR DE "PHYSICAL education for older people" OR DE "PHYSICAL fitness for older people" OR DE "SPORTS for older peo	18369
(middleage* or (middle N1 age*) or "old age" or midlife or aged or aging or ageing or elderly or elders or senior or seniors or geriatric* or ol	105906
((community or independent or solo or alone) N3 (dwelling or living))	3907
S1 or S2 or S3	107287
DE "RESISTANCE training" OR DE "CONTRAST training (Physical training & conditioning)"	3084
((power or high-velocity or velocity or ballistic or explosive*) N5 (train* or lift* or resistance or concentric or exerci*))	7147
("high-speed resistance")	8
((fast or quick or speed* or velocity) N2 (reps or repetition*))	185
("complex training" or "contrast training" or "speed-strength")	793
S5 OR S6 OR S7 OR S8 OR S9	10655
(randomized or randomised or randomly or RCT or RCT or placebo* or trial)	79383
((singl* or doubl* or trebl* or tripl*) N1 (mask* or blind* or dumm*))	7710
S11 or S12	79854
S4 and S10 and S13	409

© 2022 Balachandran AT et al. *JAMA Network Open*.

TIAB"power training" AND method "clinical trial"	49
TIAB"velocity training" AND method "clinical trial"	6
TIAB"ballistic training" AND method "clinical trial"	1
TIAB"explosive training" AND method "clinical trial"	2
TIAB"high-speed resistance" AND method "clinical trial"	8
TIAB"complex training" AND method "clinical trial"	3
TIAB"contrast training" AND method "clinical trial"	1
TIAB"speed-strength" AND method "clinical trial"	8
Total unique	71

© 2022 Balachandran AT et al. *JAMA Network Open*.

eAppendix 2. Risk of Bias for physical function outcome

**Unique ID**	1	**Study ID**	Bottaro	**Assessor**	
**Ref or Label**	Bottaro	**Aim**	assignment to intervention (the 'intention-to-treat' effect)		
**Experimental**	Power	**Comparator**	Control	**Source**	Journal article(s)
**Outcome**	Physical Function	**Results**		**Weight**	1

Domain	Signalling question	Response	Comments
**Bias arising from the randomization process**	1.1 Was the allocation sequence random?	NI	No info about method of randomization or concealment. Just says " randomly assigned".
	1.2 Was the allocation sequence concealed until participants were enrolled and assigned to interventions?	NI	
	1.3 Did baseline differences between intervention groups suggest a problem with the randomization process?	N	NO imbalances are apparent or if any observed imbalances are compatible with chance.
	**Risk of bias judgement**	**Some concerns**	
**Bias due to deviations from intended interventions**	2.1.Were participants aware of their assigned intervention during the trial?	Y	Yes, both partcipants and interventionisits were aware of the groups
	2.2.Were carers and people delivering the interventions aware of participants' assigned intervention during the trial?	Y	
	2.3. If Y/PY/NI to 2.1 or 2.2: Were there deviations from the intended intervention that arose because of the experimental context?	PN	The control group was exercising too.They were exercising at a center. Deviations due to trial context are very unlikely.
	2.4 If Y/PY to 2.3: Were these deviations likely to have affected the outcome?	NA	
	2.5. If Y/PY/NI to 2.4: Were these deviations from intended intervention balanced between groups?	NA	
	2.6 Was an appropriate analysis used to estimate the effect of assignment to intervention?	Y	Yes. They used a Modified ITT. The researchers didn't exclude anyone nor were anyone analyzed in the wrong group.
	2.7 If N/PN/NI to 2.6: Was there potential for a substantial impact (on the result) of the failure to analyse participants in the group to which they were randomized?	NA	
	**Risk of bias judgement**	**Low**	
**Bias due to missing outcome data**	3.1 Were data for this outcome available for all, or nearly all, participants randomized?	N	16% dropped out (< 95% fr continous outcomes). 2 dropped for PT group and 3 from Control due to "family and personal reasons".
	3.2 If N/PN/NI to 3.1: Is there evidence that result was not biased by missing outcome data?	N	No they did not perform any imputation or sensitivty analysis.
	3.3 If N/PN to 3.2: Could missingness in the outcome depend on its true value?	PN	No. Although differential drop-out, reasons were due to family and personal reasons and hence unrelated to the outcome . Also greater drops outs in the control group than intervention.
	3.4 If Y/PY/NI to 3.3: Is it likely that missingness in the outcome depended on its true value?	NA	
	**Risk of bias judgement**	**Low**	
**Bias in measurement of the outcome**	4.1 Was the method of measuring the outcome inappropriate?	N	Had validated and senitive measures.
	4.2 Could measurement or ascertainment of the outcome have differed between intervention groups?	N	outcome measurement (data collection) involve the same measurement methods and thresholds, used at comparable time points
	4.3 Were outcome assessors aware of the intervention received by study participants?	N	Assessors ere blinded is not reported in the paper. But email from author says single blind. .
	4.4 If Y/PY/NI to 4.3: Could assessment of the outcome have been influenced by knowledge of intervention received?	NA	
	4.5 If Y/PY/NI to 4.4: Is it likely that assessment of the outcome was influenced by knowledge of intervention received?	NA	
	**Risk of bias judgement**	**Low**	
**Bias in selection of the reported result**	5.1 Were the data that produced this result analysed in accordance with a pre-specified analysis plan that was finalized before unblinded outcome data were available for analysis?	NI	No pre-specified analysis or protocol mentioned
	5.2 ... multiple eligible outcome measurements (e.g. scales, definitions, time points) within the outcome domain?	N	Standard scales used at final time point. Reported functional outcomes. For ex, chair stand and get up and go required for meta.
	5.3 ... multiple eligible analyses of the data?	N	performed using a 2* 2 (between-within) analysis of variance [time (preand post-test) £ group (PT and TRT)] with a least-significant diVerence (LSD) post-hoc procedure. Reported raw post values. .
	**Risk of bias judgement**	**Low**	
**Overall bias**	**Risk of bias judgement**	**Some concerns**	

**Unique ID**	2	**Study ID**	Correa	**Assessor**	
**Ref or Label**	Correa	**Aim**	assignment to intervention (the 'intention-to-treat' effect)		
**Experimental**	Power	**Comparator**	Control	**Source**	
**Outcome**	Physical Function	**Results**		**Weight**	1

Domain	Signalling question	Response	Comments
**Bias arising from the randomization process**	1.1 Was the allocation sequence random?	NI	No info about method of randomization or concealment. Just says " randomly assigned".
	1.2 Was the allocation sequence concealed until participants were enrolled and assigned to interventions?	NI	
	1.3 Did baseline differences between intervention groups suggest a problem with the randomization process?	PN	NO imbalances are apparent or if any observed imbalances are compatible with chance.
	**Risk of bias judgement**	**Some concerns**	

© 2022 Balachandran AT et al. *JAMA Network Open*.

**Bias due to deviations from intended interventions**	2.1.Were participants aware of their assigned intervention during the trial?	Y	Partcipants and Interventionists were aware.
	2.2.Were carers and people delivering the interventions aware of participants' assigned intervention during the trial?	PY	
	2.3. If Y/PY/NI to 2.1 or 2.2: Were there deviations from the intended intervention that arose because of the experimental context?	N	The control group was exercising too.They were exercising at a center. Deviations due to trial context are very unlikely.
	2.4 If Y/PY to 2.3: Were these deviations likely to have affected the outcome?	NA	
	2.5. If Y/PY/NI to 2.4: Were these deviations from intended intervention balanced between groups?	NA	
	2.6 Was an appropriate analysis used to estimate the effect of assignment to intervention?	Y	Yes. They used a Modified ITT. The researchers didn't exclude anyone nor were anyone analyzed in the wrong group.
	2.7 If N/PN/NI to 2.6: Was there potential for a substantial impact (on the result) of the failure to analyse participants in the group to which they were randomized?	NA	
	**Risk of bias judgement**	**Low**	
**Bias due to missing outcome data**	3.1 Were data for this outcome available for all, or nearly all, participants randomized?	Y	No drop outs reported in the final 6 weeks
	3.2 If N/PN/NI to 3.1: Is there evidence that result was not biased by missing outcome data?	NA	
	3.3 If N/PN to 3.2: Could missingness in the outcome depend on its true value?	NA	
	3.4 If Y/PY/NI to 3.3: Is it likely that missingness in the outcome depended on its true value?	NA	
	**Risk of bias judgement**	**Low**	
**Bias in measurement of the outcome**	4.1 Was the method of measuring the outcome inappropriate?	N	Valid meaures
	4.2 Could measurement or ascertainment of the outcome have differed between intervention groups?	PN	outcome measurement (data collection) involve the same measurement methods and thresholds, used at comparable time points
	4.3 Were outcome assessors aware of the intervention received by study participants?	N	Author email said "evaluators were blinded"(S Correa)
	4.4 If Y/PY/NI to 4.3: Could assessment of the outcome have been influenced by knowledge of intervention received?	NA	
	4.5 If Y/PY/NI to 4.4: Is it likely that assessment of the outcome was influenced by knowledge of intervention received?	NA	
	**Risk of bias judgement**	**Low**	Changed to some concerns since we are unsure ofthe blinding. No reponse from authors.
**Bias in selection of the reported result**	5.1 Were the data that produced this result analysed in accordance with a pre-specified analysis plan that was finalized before unblinded outcome data were available for analysis?	NI	No pre-specified analysis or protocol menotioned
	5.2 ... multiple eligible outcome measurements (e.g. scales, definitions, time points) within the outcome domain?	N	Just post data at 12 weeks. Reported functional outcomes. For ex, chair stand required for meta.
	5.3 ... multiple eligible analyses of the data?	N	2-way repeated measures analysis of variance (ANOVA) was used (2 groups × 3 times), with Bonferroni post-hoc tests. Reported raw post scores
	**Risk of bias judgement**	**Low**	
**Overall bias**	**Risk of bias judgement**	**Some concerns**	

**Unique ID**	3	**Study ID**	Zech	**Assessor**	
**Ref or Label**	Zech	**Aim**	assignment to intervention (the 'intention-to-treat' effect)		
**Experimental**	Power	**Comparator**	Control	**Source**	Journal article(s)
**Outcome**	Physical Function	**Results**		**Weight**	1

**Domain**	**Signalling question**	**Response**	**Comments**
**Bias arising from the randomization process**	1.1 Was the allocation sequence random?	Y	Yes. " by a researcher not involved in this study. Randomization was computer-generated in blocks of 12–15 participants and the blinded assessor handed out sealed envelopes with group assignment to each participant"
	1.2 Was the allocation sequence concealed until participants were enrolled and assigned to interventions?	Y	
	1.3 Did baseline differences between intervention groups suggest a problem with the randomization process?	N	No major differences
	**Risk of bias judgement**	**Low**	
**Bias due to deviations from intended interventions**	2.1.Were participants aware of their assigned intervention during the trial?	Y	Both were aware.
	2.2.Were carers and people delivering the interventions aware of participants' assigned intervention during the trial?	Y	
	2.3. If Y/PY/NI to 2.1 or 2.2: Were there deviations from the intended intervention that arose because of the experimental context?	PN	The control group was exercising too.They were exercising at a center and supervised. Deviations due to trial context are very unlikely.
	2.4 If Y/PY to 2.3: Were these deviations likely to have affected the outcome?	NA	
	2.5. If Y/PY/NI to 2.4: Were these deviations from intended intervention balanced between groups?	NA	
	2.6 Was an appropriate analysis used to estimate the effect of assignment to intervention?	Y	Used a MIIT
	2.7 If N/PN/NI to 2.6: Was there potential for a substantial impact (on the result) of the failure to analyse participants in the group to which they were randomized?	NA	
	**Risk of bias judgement**	**Low**	
	3.1 Were data for this outcome available for all, or nearly all, participants randomized?	N	25% dropped in Power and 15% in Control. 19% in total. 2 dropped due to exacerbation of arthritis and vertigo from the power group.

© 2022 Balachandran AT et al. *JAMA Network Open*.

**Bias due to missing outcome data**	3.2 If N/PN/NI to 3.1: Is there evidence that result was not biased by missing outcome data?	N	No sensitivty or imutations perfomed for missing outcome.
	3.3 If N/PN to 3.2: Could missingness in the outcome depend on its true value?	PY	Although differential drop-out, only 2 drop-outs related to outcome (10%) and the rest unrelated.
	3.4 If Y/PY/NI to 3.3: Is it likely that missingness in the outcome depended on its true value?	PN	
	**Risk of bias judgement**	**Some concerns**	
**Bias in measurement of the outcome**	4.1 Was the method of measuring the outcome inappropriate?	N	Yes. valid measures.
	4.2 Could measurement or ascertainment of the outcome have differed between intervention groups?	N	outcome measurement (data collection) involve the same measurement methods and thresholds, used at comparable time points
	4.3 Were outcome assessors aware of the intervention received by study participants?	PN	Assessors being blind reported in pre-reg , but not mentioned in paper
	4.4 If Y/PY/NI to 4.3: Could assessment of the outcome have been influenced by knowledge of intervention received?	NA	
	4.5 If Y/PY/NI to 4.4: Is it likely that assessment of the outcome was influenced by knowledge of intervention received?	NA	
	**Risk of bias judgement**	**Low**	
**Bias in selection of the reported result**	5.1 Were the data that produced this result analysed in accordance with a pre-specified analysis plan that was finalized before unblinded outcome data were available for analysis?	Y	Preregistration available, but not SAP
	5.2 ... multiple eligible outcome measurements (e.g. scales, definitions, time points) within the outcome domain?	PN	Final time point and SPPB. Reported functional outcomes , SPBB required for meta.
	5.3 ... multiple eligible analyses of the data?	PN	A two factorial linear mixed model, appropriate for repeated measures data, was used to analyze continuous data in the main and secondary outcome variables. Reported post scores for SPPB.
	**Risk of bias judgement**	**Low**	
**Overall bias**	**Risk of bias judgement**	**Some concerns**	

**Unique ID**	4	**Study ID**	Henwood	**Assessor**	
**Ref or Label**	Henwood	**Aim**	assignment to intervention (the 'intention-to-treat' effect)		
**Experimental**	Power	**Comparator**	Control	**Source**	Journal article(s)
**Outcome**	Physical Function	**Results**		**Weight**	1

**Domain**	**Signalling question**	**Response**	**Comments**
**Bias arising from the randomization process**	1.1 Was the allocation sequence random?	NI	NI about randomization or conealment. Just wrote "randomized to either "
	1.2 Was the allocation sequence concealed until participants were enrolled and assigned to interventions?	NI	
	1.3 Did baseline differences between intervention groups suggest a problem with the randomization process?	N	No major baseline differences
	**Risk of bias judgement**	**Some concerns**	
**Bias due to deviations from intended interventions**	2.1.Were participants aware of their assigned intervention during the trial?	Y	Yes, both participants and interventionists were aware of the groups
	2.2.Were carers and people delivering the interventions aware of participants' assigned intervention during the trial?	PY	
	2.3. If Y/PY/NI to 2.1 or 2.2: Were there deviations from the intended intervention that arose because of the experimental context?	PN	The control group was exercising too. They were exercising at a center. Deviations due to trial context are very unlikely.
	2.4 If Y/PY to 2.3: Were these deviations likely to have affected the outcome?	NA	
	2.5. If Y/PY/NI to 2.4: Were these deviations from intended intervention balanced between groups?	NA	
	2.6 Was an appropriate analysis used to estimate the effect of assignment to intervention?	Y	Yes. They used a Modified ITT. The researchers didn't exclude anyone nor were anyone analyzed in the wrong group.
	2.7 If N/PN/NI to 2.6: Was there potential for a substantial impact (on the result) of the failure to analyse participants in the group to which they were randomized?	NA	
	**Risk of bias judgement**	**Low**	
**Bias due to missing outcome data**	3.1 Were data for this outcome available for all, or nearly all, participants randomized?	PN	4 dropped and 3 from the control. 15% drop out.
	3.2 If N/PN/NI to 3.1: Is there evidence that result was not biased by missing outcome data?	PN	No No they did not perform any imputation or sensitivty analysis.
	3.3 If N/PN to 3.2: Could missingness in the outcome depend on its true value?	PN	No. Although differential drop-out, "no participants indicated that the training protocol or intensity was the reason for leaving the study" . So missing likely not related to the outcome
	3.4 If Y/PY/NI to 3.3: Is it likely that missingness in the outcome depended on its true value?	NA	
	**Risk of bias judgement**	**Low**	
**Bias in measurement of the outcome**	4.1 Was the method of measuring the outcome inappropriate?	N	Outcome measurement (data collection) involve the same measurement methods and thresholds, used at comparable time points
	4.2 Could measurement or ascertainment of the outcome have differed between intervention groups?	N	
	4.3 Were outcome assessors aware of the intervention received by study participants?	NI	No information about blinding
	4.4 If Y/PY/NI to 4.3: Could assessment of the outcome have been influenced by knowledge of intervention received?	PY	Yes , outcome involves use of a stop watch and verbal encouragement can affect outcome. We are unsure if blinding was implemented, hence we rated as " some concerns"
	4.5 If Y/PY/NI to 4.4: Is it likely that assessment of the outcome was influenced by knowledge of intervention received?	PN	

© 2022 Balachandran AT et al. *JAMA Network Open*.

	**Risk of bias judgement**	**Some concerns**	
**Bias in selection of the reported result**	5.1 Were the data that produced this result analysed in accordance with a pre-specified analysis plan that was finalized before unblinded outcome data were available for analysis?	NI	No pre-specified analysis or protocol mentioned
	5.2 ... multiple eligible outcome measurements (e.g. scales, definitions, time points) within the outcome domain?	PN	Standard scales used at one time point. Reported functional outcomes. For ex, chair stand and stair climb and go required for meta.
	5.3 ... multiple eligible analyses of the data?	PN	a two-way (Group * Time) repeated-measures analysis of covariance (ANCOVA) adjusted for sex. Adjusted scores given and not raw scores.
	**Risk of bias judgement**	**Some concerns**	
**Overall bias**	**Risk of bias judgement**	**Some concerns**	

**Unique ID**	5	**Study ID**	Marsh	**Assessor**	
**Ref or Label**	Marsh	**Aim**	assignment to intervention (the 'intention-to-treat' effect)		
**Experimental**		**Comparator**		**Source**	Journal article(s)
**Outcome**	Physical Function	**Results**		**Weight**	1

**Domain**	**Signalling question**	**Response**	**Comments**
**Bias arising from the randomization process**	1.1 Was the allocation sequence random?	PY	Used a computer web based sytem. "participants were assigned to treatment using a computer-generated randomization scheme integrated into a Web-based data-entry and -management system." Very likely concealed too.
	1.2 Was the allocation sequence concealed until participants were enrolled and assigned to interventions?	PY	
	1.3 Did baseline differences between intervention groups suggest a problem with the randomization process?	N	No imbalances are apparent or observed imbalances are compatible with chance.
	**Risk of bias judgement**	**Low**	
**Bias due to deviations from intended interventions**	2.1.Were participants aware of their assigned intervention during the trial?	Y	Yes, both participants and interventionists were aware of the groups
	2.2.Were carers and people delivering the interventions aware of participants' assigned intervention during the trial?	Y	
	2.3. If Y/PY/NI to 2.1 or 2.2: Were there deviations from the intended intervention that arose because of the experimental context?	PN	The control group was exercising too. All sessions were supervised and they were exercising at a center. Deviations due to trial context are very unlikely.
	2.4 If Y/PY to 2.3: Were these deviations likely to have affected the outcome?	NA	
	2.5. If Y/PY/NI to 2.4: Were these deviations from intended intervention balanced between groups?	NA	
	2.6 Was an appropriate analysis used to estimate the effect of assignment to intervention?	Y	Yes. They used a Modified ITT. The researchers didn't exclude anyone.
	2.7 If N/PN/NI to 2.6: Was there potential for a substantial impact (on the result) of the failure to analyse participants in the group to which they were randomized?	NA	
	**Risk of bias judgement**	**Low**	
**Bias due to missing outcome data**	3.1 Were data for this outcome available for all, or nearly all, participants randomized?	N	Around 25% in I and 33% in C drop out in each group. 30% in total.
	3.2 If N/PN/NI to 3.1: Is there evidence that result was not biased by missing outcome data?	PN	No they did not perform any imputation or sensitivty analysis
	3.3 If N/PN to 3.2: Could missingness in the outcome depend on its true value?	PN	Missing was not related to true value. More dropped from control. None of the drop outs were related to the intervention. " all AEs and SAEs and did not attribute any of them to the interventions."
	3.4 If Y/PY/NI to 3.3: Is it likely that missingness in the outcome depended on its true value?	NA	
	**Risk of bias judgement**	**Low**	
**Bias in measurement of the outcome**	4.1 Was the method of measuring the outcome inappropriate?	N	Outcome measurement (data collection) involve the same measurement methods and thresholds, used at comparable time points
	4.2 Could measurement or ascertainment of the outcome have differed between intervention groups?	N	
	4.3 Were outcome assessors aware of the intervention received by study participants?	N	Asesors blinded to group outcomes. Emailed by the author.
	4.4 If Y/PY/NI to 4.3: Could assessment of the outcome have been influenced by knowledge of intervention received?	NA	
	4.5 If Y/PY/NI to 4.4: Is it likely that assessment of the outcome was influenced by knowledge of intervention received?	NA	
	**Risk of bias judgement**	**Low**	
**Bias in selection of the reported result**	5.1 Were the data that produced this result analysed in accordance with a pre-specified analysis plan that was finalized before unblinded outcome data were available for analysis?	NI	No protocol or registration.
	5.2 ... multiple eligible outcome measurements (e.g. scales, definitions, time points) within the outcome domain?	PN	Standard scales used at one time point. Reported functional outcomes. For ex, SPPB required for meta.
	5.3 ... multiple eligible analyses of the data?	PN	ANCOVA controlling for the pretest score used. Howevere, unadjusted score reported.
	**Risk of bias judgement**	**Low**	
**Overall bias**	**Risk of bias judgement**	**Low**	

**Unique ID**	6	**Study ID**	Gray	**Assessor**	

© 2022 Balachandran AT et al. *JAMA Network Open*.

**Ref or Label**		**Aim**	assignment to intervention (the 'intention-to-treat' effect)		
**Experimental**	Power	**Comparator**	Control	**Source**	
**Outcome**	Physical function	**Results**		**Weight**	1

Domain	Signalling question	Response	Comments
**Bias arising from the randomization process**	1.1 Was the allocation sequence random?	Y	NI about concealment. Just mentioned" randomly assigned to one of three"..using a random numbers generator."
	1.2 Was the allocation sequence concealed until participants were enrolled and assigned to interventions?	NI	
	1.3 Did baseline differences between intervention groups suggest a problem with the randomization process?	PY	Sample sizes for 3 groups very different. (41,34 and 24)
	**Risk of bias judgement**	**High**	
**Bias due to deviations from intended interventions**	2.1.Were participants aware of their assigned intervention during the trial?	Y	Yes, both participants and interventionists were aware of the groups
	2.2.Were carers and people delivering the interventions aware of participants' assigned intervention during the trial?	PY	
	2.3. If Y/PY/NI to 2.1 or 2.2: Were there deviations from the intended intervention that arose because of the experimental context?	PN	The control group was exercising too. They were exercising at a communty center. Deviations due to trial context are very unlikely. " supervised by a member of the research team
	2.4 If Y/PY to 2.3: Were these deviations likely to have affected the outcome?	NA	
	2.5. If Y/PY/NI to 2.4: Were these deviations from intended intervention balanced between groups?	NA	
	2.6 Was an appropriate analysis used to estimate the effect of assignment to intervention?	PY	Yes. They used a Modified ITT. The researchers didn't exclude anyone nor were anyone analyzed in the wrong group.
	2.7 If N/PN/NI to 2.6: Was there potential for a substantial impact (on the result) of the failure to analyse participants in the group to which they were randomized?	NA	
	**Risk of bias judgement**	**Low**	
**Bias due to missing outcome data**	3.1 Were data for this outcome available for all, or nearly all, participants randomized?	N	
	3.2 If N/PN/NI to 3.1: Is there evidence that result was not biased by missing outcome data?	PN	No they did not perform any imputation or sensitivty analysis.
	3.3 If N/PN to 3.2: Could missingness in the outcome depend on its true value?	PN	There was a 17% differemce in drop outs betwen the groups. But The most common reasons for dropping out were lack of interest, health issues, and scheduling conflicts. Highest drop out was int he control group.
	3.4 If Y/PY/NI to 3.3: Is it likely that missingness in the outcome depended on its true value?	NA	
	**Risk of bias judgement**	**Low**	
**Bias in measurement of the outcome**	4.1 Was the method of measuring the outcome inappropriate?	PN	Had validated and sensitive measures.
	4.2 Could measurement or ascertainment of the outcome have differed between intervention groups?	PN	Outcome measurement (data collection) involve the same measurement methods and thresholds, used at comparable time points
	4.3 Were outcome assessors aware of the intervention received by study participants?	NI	
	4.4 If Y/PY/NI to 4.3: Could assessment of the outcome have been influenced by knowledge of intervention received?	PY	Yes , outcome involves use of a stop watch and verbal encouragement can affect outcome. We are unsure if blinding was implemented, hence we rated as "some concerns"
	4.5 If Y/PY/NI to 4.4: Is it likely that assessment of the outcome was influenced by knowledge of intervention received?	PN	
	**Risk of bias judgement**	**Some concerns**	
**Bias in selection of the reported result**	5.1 Were the data that produced this result analysed in accordance with a pre-specified analysis plan that was finalized before unblinded outcome data were available for analysis?	NI	No pre-specified analysis or protocol mentioned
	5.2 ... multiple eligible outcome measurements (e.g. scales, definitions, time points) within the outcome domain?	PN	Standard scales used at one time point. Reported functional outcomes. For ex, chair stand and get up and go required for meta
	5.3 ... multiple eligible analyses of the data?	PN	Used ANCOVA adjusted for baseline. Unsure about if adjusted scores.
	**Risk of bias judgement**	**Some concerns**	
**Overall bias**	**Risk of bias judgement**	**High**	

**Unique ID**	7	**Study ID**	Lopez	**Assessor**	
**Ref or Label**		**Aim**	assignment to intervention (the 'intention-to-treat' effect)		
**Experimental**	Power	**Comparator**	Control	**Source**	Journal article(s)
**Outcome**	Physical function	**Results**		**Weight**	1

Domain	Signalling question	Response	Comments
**Bias arising from the randomization process**	1.1 Was the allocation sequence random?	PY	No info about method of randomization or concealment. Just says " were randomly assigned (using a computer generated list) to 2"
	1.2 Was the allocation sequence concealed until participants were enrolled and assigned to interventions?	NI	
	1.3 Did baseline differences between intervention groups suggest a problem with the randomization process?	PN	No imbalances are apparent or observed imbalances are compatible with chance.
	**Risk of bias judgement**	**Some concerns**	
	2.1.Were participants aware of their assigned intervention during the trial?	Y	Yes, both participants and interventionists were aware of the groups

© 2022 Balachandran AT et al. *JAMA Network Open*.

Bias due to deviations from intended interventions	2.2.Were carers and people delivering the interventions aware of participants' assigned intervention during the trial?	Y	
	2.3. If Y/PY/NI to 2.1 or 2.2: Were there deviations from the intended intervention that arose because of the experimental context?	PN	The control group was exercising too. They were exercising at a center. Deviations due to trial context are very unlikely. "performed under the direct supervision of an exercise instructor to ensure safety and the maintenance of
	2.4 If Y/PY to 2.3: Were these deviations likely to have affected the outcome?	NA	
	2.5. If Y/PY/NI to 2.4: Were these deviations from intended intervention balanced between groups?	NA	
	2.6 Was an appropriate analysis used to estimate the effect of assignment to intervention?	PY	Yes. They used a Modified ITT. The researchers didn't exclude anyone nor were anyone analyzed in the wrong group.
	2.7 If N/PN/NI to 2.6: Was there potential for a substantial impact (on the result) of the failure to analyse participants in the group to which they were randomized?	NA	
	**Risk of bias judgement**	**Low**	
Bias due to missing outcome data	3.1 Were data for this outcome available for all, or nearly all, participants randomized?	PN	8 dropped from Int. and 6 from control. So 10% difference
	3.2 If N/PN/NI to 3.1: Is there evidence that result was not biased by missing outcome data?	N	No, they did not perform any imputation or sensitivty analysis.
	3.3 If N/PN to 3.2: Could missingness in the outcome depend on its true value?	NI	No info about why they dropped out. Also int showed sig. improvement in chair stand and TUG.
	3.4 If Y/PY/NI to 3.3: Is it likely that missingness in the outcome depended on its true value?	NI	
	**Risk of bias judgement**	**High**	
Bias in measurement of the outcome	4.1 Was the method of measuring the outcome inappropriate?	PN	Had validated and sensitive measures
	4.2 Could measurement or ascertainment of the outcome have differed between intervention groups?	PN	Outcome measurement (data collection) involve the same measurement methods and thresholds, used at comparable time points
	4.3 Were outcome assessors aware of the intervention received by study participants?	N	Blinded based on authos email "Yes, it was a single blind study."
	4.4 If Y/PY/NI to 4.3: Could assessment of the outcome have been influenced by knowledge of intervention received?	NA	
	4.5 If Y/PY/NI to 4.4: Is it likely that assessment of the outcome was influenced by knowledge of intervention received?	NA	
	**Risk of bias judgement**	**Low**	
Bias in selection of the reported result	5.1 Were the data that produced this result analysed in accordance with a pre-specified analysis plan that was finalized before unblinded outcome data were available for analysis?	NI	No pre-specified analysis or protocol mentioned
	5.2 ... multiple eligible outcome measurements (e.g. scales, definitions, time points) within the outcome domain?	PN	Standard scales used at one time point. Reported functional outcomes. For ex, chair stand and get up and go required for meta.
	5.3 ... multiple eligible analyses of the data?	PN	unadjusted extracted from graphs
	**Risk of bias judgement**	**Low**	
**Overall bias**	**Risk of bias judgement**	**High**	

**Unique ID**	8	**Study ID**	Richardson	**Assessor**	
**Ref or Label**		**Aim**	assignment to intervention (the 'intention-to-treat' effect)		
**Experimental**	Power	**Comparator**	Control	**Source**	
**Outcome**	Physical Function	**Results**		**Weight**	1

Domain	Signalling question	Response	Comments
Bias arising from the randomization process	1.1 Was the allocation sequence random?	PY	No info about method of randomization or concealment. Just says " randomly allocated".
	1.2 Was the allocation sequence concealed until participants were enrolled and assigned to interventions?	NI	
	1.3 Did baseline differences between intervention groups suggest a problem with the randomization process?	PN	No imbalances are apparent or observed imbalances are compatible with chance.
	**Risk of bias judgement**	**Some concerns**	
Bias due to deviations from intended interventions	2.1.Were participants aware of their assigned intervention during the trial?	Y	Yes, both participants and interventionists were aware of the groups
	2.2.Were carers and people delivering the interventions aware of participants' assigned intervention during the trial?	PY	
	2.3. If Y/PY/NI to 2.1 or 2.2: Were there deviations from the intended intervention that arose because of the experimental context?	PN	The control group was exercising too. They were exercising at a center. Deviations due to trial context are very unlikely.
	2.4 If Y/PY to 2.3: Were these deviations likely to have affected the outcome?	NA	
	2.5. If Y/PY/NI to 2.4: Were these deviations from intended intervention balanced between groups?	NA	
	2.6 Was an appropriate analysis used to estimate the effect of assignment to intervention?	PY	Yes. They used a Modified ITT. The researchers didn't exclude anyone nor were anyone analyzed in the wrong group.
	2.7 If N/PN/NI to 2.6: Was there potential for a substantial impact (on the result) of the failure to analyse participants in the group to which they were randomized?	NA	
	**Risk of bias judgement**	**Low**	
	3.1 Were data for this outcome available for all, or nearly all, participants randomized?	PY	only 1 drop put 5%
	3.2 If N/PN/NI to 3.1: Is there evidence that result was not biased by missing outcome data?	NA	

© 2022 Balachandran AT et al. *JAMA Network Open*.

Bias due to missing outcome data	3.3 If N/PN to 3.2: Could missingness in the outcome depend on its true value?	NA	
	3.4 If Y/PY/NI to 3.3: Is it likely that missingness in the outcome depended on its true value?	NA	
	**Risk of bias judgement**	**Low**	
Bias in measurement of the outcome	4.1 Was the method of measuring the outcome inappropriate?	N	Had validated and sensitive measures.
	4.2 Could measurement or ascertainment of the outcome have differed between intervention groups?	PN	Outcome measurement (data collection) involve the same measurement methods and thresholds, used at comparable time points
	4.3 Were outcome assessors aware of the intervention received by study participants?	Y	Assessors were not blind to groups stated in paper
	4.4 If Y/PY/NI to 4.3: Could assessment of the outcome have been influenced by knowledge of intervention received?	PY	Yes , outcome involves use of a stop watch and verbal encouragement can affect outcome.
	4.5 If Y/PY/NI to 4.4: Is it likely that assessment of the outcome was influenced by knowledge of intervention received?	PY	
	**Risk of bias judgement**	**High**	
Bias in selection of the reported result	5.1 Were the data that produced this result analysed in accordance with a pre-specified analysis plan that was finalized before unblinded outcome data were available for analysis?	NI	No pre-specified analysis or protocol mentioned
	5.2 ... multiple eligible outcome measurements (e.g. scales, definitions, time points) within the outcome domain?	PN	Standard scales used at one time point. Reported functional outcomes. For ex, chair stabd and 8 ft up and go required for meta.
	5.3 ... multiple eligible analyses of the data?	PN	ANCOVA performed. But unadjusted scores reported. TUG not significant
	**Risk of bias judgement**	**Low**	
**Overall bias**	**Risk of bias judgement**	**High**	

**Unique ID**	9	**Study ID**	Tiggermann	**Assessor**	
**Ref or Label**		**Aim**	assignment to intervention (the 'intention-to-treat' effect)		
**Experimental**	Power	**Comparator**	Control	**Source**	Journal article(s)
**Outcome**		**Results**		**Weight**	1

Domain	Signalling question	Response	Comments
Bias arising from the randomization process	1.1 Was the allocation sequence random?	PY	No info about method of randomization or concealment. Just says " randomly assigned. " Participants were randomly assigned"
	1.2 Was the allocation sequence concealed until participants were enrolled and assigned to interventions?	NI	
	1.3 Did baseline differences between intervention groups suggest a problem with the randomization process?	PN	No imbalances are apparent or observed imbalances are compatible with chance.
	**Risk of bias judgement**	**Some concerns**	
Bias due to deviations from intended interventions	2.1.Were participants aware of their assigned intervention during the trial?	Y	Yes, both participants and interventionists were aware of the groups
	2.2.Were carers and people delivering the interventions aware of participants' assigned intervention during the trial?	PY	
	2.3. If Y/PY/NI to 2.1 or 2.2: Were there deviations from the intended intervention that arose because of the experimental context?	PN	The control group was exercising too. They were exercising at a center and supervised. Deviations due to trial context are very unlikely
	2.4 If Y/PY to 2.3: Were these deviations likely to have affected the outcome?	NA	
	2.5. If Y/PY/NI to 2.4: Were these deviations from intended intervention balanced between groups?	NA	
	2.6 Was an appropriate analysis used to estimate the effect of assignment to intervention?	PY	Yes. They used a Modified ITT. The researchers didn't exclude anyone nor were anyone analyzed in the wrong group.
	2.7 If N/PN/NI to 2.6: Was there potential for a substantial impact (on the result) of the failure to analyse participants in the group to which they were randomized?	NA	
	**Risk of bias judgement**	**Low**	
Bias due to missing outcome data	3.1 Were data for this outcome available for all, or nearly all, participants randomized?	PN	20% (3) in PT and 13% (2) in each group
	3.2 If N/PN/NI to 3.1: Is there evidence that result was not biased by missing outcome data?	PN	No they did not perform any imputation or sensitivty analysis.
	3.3 If N/PN to 3.2: Could missingness in the outcome depend on its true value?	PN	1 discontuned intervention but no reasons given. 1 unrelated to study. So 2 not accounted. So some concerns. 2 Drop puts in control group unrleated to study. No sig difference in function scores.
	3.4 If Y/PY/NI to 3.3: Is it likely that missingness in the outcome depended on its true value?	NA	
	**Risk of bias judgement**	**Some concerns**	
Bias in measurement of the outcome	4.1 Was the method of measuring the outcome inappropriate?	PN	Had validated and sensitive measures.
	4.2 Could measurement or ascertainment of the outcome have differed between intervention groups?	PN	Outcome measurement (data collection) involve the same measurement methods and thresholds, used at comparable time points
	4.3 Were outcome assessors aware of the intervention received by study participants?	N	single blind, but sure what they mean
	4.4 If Y/PY/NI to 4.3: Could assessment of the outcome have been influenced by knowledge of intervention received?	NA	
	4.5 If Y/PY/NI to 4.4: Is it likely that assessment of the outcome was influenced by knowledge of intervention received?	NA	
	**Risk of bias judgement**	**Low**	

© 2022 Balachandran AT et al. *JAMA Network Open*.

Bias in selection of the reported result	5.1 Were the data that produced this result analysed in accordance with a pre-specified analysis plan that was finalized before unblinded outcome data were available for analysis?	NI	No pre-specified analysis or protocol mentioned
	5.2 ... multiple eligible outcome measurements (e.g. scales, definitions, time points) within the outcome domain?	PN	Standard scales used at one time point. Reported functional outcomes. For ex, chair stand and stair climb required for meta.
	5.3 ... multiple eligible analyses of the data?	N	Training-related effects were assessed using a two-way analysis of variance (ANOVA) with repeated measures (group vs. time). Unadjusted scores reported.
	**Risk of bias judgement**	**Low**	
**Overall bias**	**Risk of bias judgement**	**Some concerns**	

**Unique ID**	10	**Study ID**	Jaque	**Assessor**	
**Ref or Label**		**Aim**	assignment to intervention (the 'intention-to-treat' effect)		
**Experimental**	Power	**Comparator**	Control	**Source**	Journal article(s)
**Outcome**	Physical Function	**Results**		**Weight**	1

Domain	Signalling question	Response	Comments
Bias arising from the randomization process	1.1 Was the allocation sequence random?	PY	No info about method of randomization or concealment. Just says " randomly allocated"
	1.2 Was the allocation sequence concealed until participants were enrolled and assigned to interventions?	NI	
	1.3 Did baseline differences between intervention groups suggest a problem with the randomization process?	PN	No imbalances are apparent or observed imbalances are compatible with chance.
	**Risk of bias judgement**	**Some concerns**	
Bias due to deviations from intended interventions	2.1.Were participants aware of their assigned intervention during the trial?	Y	Yes, both participants and interventionists were aware of the groups
	2.2.Were carers and people delivering the interventions aware of participants' assigned intervention during the trial?	PY	
	2.3. If Y/PY/NI to 2.1 or 2.2: Were there deviations from the intended intervention that arose because of the experimental context?	PN	The control group was exercising too. They were exercising at a center. Deviations due to trial context are very unlikely
	2.4 If Y/PY to 2.3: Were these deviations likely to have affected the outcome?	NA	
	2.5. If Y/PY/NI to 2.4: Were these deviations from intended intervention balanced between groups?	NA	
	2.6 Was an appropriate analysis used to estimate the effect of assignment to intervention?	PN	Excluded participants . nine females were excluded from final analyses, resulting in 14 and 12 participants completing HST and MST. Not an ITT
	2.7 If N/PN/NI to 2.6: Was there potential for a substantial impact (on the result) of the failure to analyse participants in the group to which they were randomized?	PY	
	**Risk of bias judgement**	**High**	
Bias due to missing outcome data	3.1 Were data for this outcome available for all, or nearly all, participants randomized?	Y	No drop outs or lost tof ollow ups based on the personal email from author. (Jorge)
	3.2 If N/PN/NI to 3.1: Is there evidence that result was not biased by missing outcome data?	NA	
	3.3 If N/PN to 3.2: Could missingness in the outcome depend on its true value?	NA	
	3.4 If Y/PY/NI to 3.3: Is it likely that missingness in the outcome depended on its true value?	NA	
	**Risk of bias judgement**	**Low**	
Bias in measurement of the outcome	4.1 Was the method of measuring the outcome inappropriate?	N	Had validated and sensitive measures.
	4.2 Could measurement or ascertainment of the outcome have differed between intervention groups?	PN	Outcome measurement (data collection) involve the same measurement methods and thresholds, used at comparable time points
	4.3 Were outcome assessors aware of the intervention received by study participants?	N	"The same investigator, who was blinded to the group allocation, conducted all measurements both before and after the intervention"
	4.4 If Y/PY/NI to 4.3: Could assessment of the outcome have been influenced by knowledge of intervention received?	NA	
	4.5 If Y/PY/NI to 4.4: Is it likely that assessment of the outcome was influenced by knowledge of intervention received?	NA	
	**Risk of bias judgement**	**Low**	
Bias in selection of the reported result	5.1 Were the data that produced this result analysed in accordance with a pre-specified analysis plan that was finalized before unblinded outcome data were available for analysis?	NI	No pre-specified analysis or protocol mentioned
	5.2 ... multiple eligible outcome measurements (e.g. scales, definitions, time points) within the outcome domain?	N	Standard scales used at one time point. Reported functional outcomes. For ex, get up and go required for meta. Reported post scores
	5.3 ... multiple eligible analyses of the data?	N	Yes. But reported post scores.
	**Risk of bias judgement**	**Low**	
**Overall bias**	**Risk of bias judgement**	**High**	

**Unique ID**	11	**Study ID**	Coelho-Júnior	**Assessor**	
**Ref or Label**	Coelho-Júnior	**Aim**	assignment to intervention (the 'intention-to-treat' effect)		

© 2022 Balachandran AT et al. *JAMA Network Open*.

**Experimental**	Power	**Comparator**	Control	**Source**	
**Outcome**	Physical function	**Results**		**Weight**	1

Domain	Signalling question	Response	Comments
**Bias arising from the randomization process**	1.1 Was the allocation sequence random?	Y	"A computer-generated list of random numbers was used by an independent researcher". So it likely that it is concealed.
	1.2 Was the allocation sequence concealed until participants were enrolled and assigned to interventions?	NI	
	1.3 Did baseline differences between intervention groups suggest a problem with the randomization process?	PN	Baseline difference exist.
	**Risk of bias judgement**	**Some concerns**	
**Bias due to deviations from intended interventions**	2.1.Were participants aware of their assigned intervention during the trial?	Y	
	2.2.Were carers and people delivering the interventions aware of participants' assigned intervention during the trial?	Y	
	2.3. If Y/PY/NI to 2.1 or 2.2: Were there deviations from the intended intervention that arose because of the experimental context?	PN	The control group was exercising too. They were exercising at a center. Deviations due to trial context are very unlikely
	2.4 If Y/PY to 2.3: Were these deviations likely to have affected the outcome?	NA	
	2.5. If Y/PY/NI to 2.4: Were these deviations from intended intervention balanced between groups?	NA	
	2.6 Was an appropriate analysis used to estimate the effect of assignment to intervention?	PY	MITT. one from the HSRT and one from the LSRT, withdrew after 2 weeks because they were not randomized to the same exercise group
	2.7 If N/PN/NI to 2.6: Was there potential for a substantial impact (on the result) of the failure to analyse participants in the group to which they were randomized?	NA	
	**Risk of bias judgement**	**Low**	
**Bias due to missing outcome data**	3.1 Were data for this outcome available for all, or nearly all, participants randomized?	PY	2 dropped . Each from Power and control. 9%.
	3.2 If N/PN/NI to 3.1: Is there evidence that result was not biased by missing outcome data?	NA	
	3.3 If N/PN to 3.2: Could missingness in the outcome depend on its true value?	NA	
	3.4 If Y/PY/NI to 3.3: Is it likely that missingness in the outcome depended on its true value?	NA	
	**Risk of bias judgement**	**Low**	
**Bias in measurement of the outcome**	4.1 Was the method of measuring the outcome inappropriate?	PN	
	4.2 Could measurement or ascertainment of the outcome have differed between intervention groups?	PN	
	4.3 Were outcome assessors aware of the intervention received by study participants?	NI	No information about blinding
	4.4 If Y/PY/NI to 4.3: Could assessment of the outcome have been influenced by knowledge of intervention received?	PY	
	4.5 If Y/PY/NI to 4.4: Is it likely that assessment of the outcome was influenced by knowledge of intervention received?	PY	
	**Risk of bias judgement**	**Some concerns**	
**Bias in selection of the reported result**	5.1 Were the data that produced this result analysed in accordance with a pre-specified analysis plan that was finalized before unblinded outcome data were available for analysis?	PN	Retrospective pregisitration
	5.2 ... multiple eligible outcome measurements (e.g. scales, definitions, time points) within the outcome domain?	PN	Standard scales used at one time point. Reported functional outcomes. For ex, get up and go required for meta. Reported post scores
	5.3 ... multiple eligible analyses of the data?	PN	Yes. But post data reported.
	**Risk of bias judgement**	**Low**	
**Overall bias**	**Risk of bias judgement**	**Some concerns**	

**Unique ID**	12	**Study ID**	Müller	**Assessor**	
**Ref or Label**	Müller	**Aim**	assignment to intervention (the 'intention-to-treat' effect)		
**Experimental**	Power	**Comparator**		**Source**	
**Outcome**	Physical Function	**Results**		**Weight**	1

Domain	Signalling question	Response	Comments
**Bias arising from the randomization process**	1.1 Was the allocation sequence random?	Y	"participants were randomly assigned into two separate intervention groups through electronic randomization (https ://www.rando mizer .org)"
	1.2 Was the allocation sequence concealed until participants were enrolled and assigned to interventions?	PY	"Concealment was guaranteed by a researcher who was blinded with respect to the participants."
	1.3 Did baseline differences between intervention groups suggest a problem with the randomization process?	PN	NO imbalances are apparent or if any observed imbalances are compatible with chance.
	**Risk of bias judgement**	**Low**	
	2.1.Were participants aware of their assigned intervention during the trial?	Y	
	2.2.Were carers and people delivering the interventions aware of participants' assigned intervention during the trial?	Y	

© 2022 Balachandran AT et al. *JAMA Network Open*.

Bias due to deviations from intended interventions	2.3. If Y/PY/NI to 2.1 or 2.2: Were there deviations from the intended intervention that arose because of the experimental context?	PN	The control group was exercising too. They were exercising at a center. Deviations due to trial context are very unlikely
	2.4 If Y/PY to 2.3: Were these deviations likely to have affected the outcome?	NA	
	2.5. If Y/PY/NI to 2.4: Were these deviations from intended intervention balanced between groups?	NA	
	2.6 Was an appropriate analysis used to estimate the effect of assignment to intervention?	PY	Yes. They used a Modified ITT. The researchers didn't exclude anyone nor were anyone analyzed in the wrong group.
	2.7 If N/PN/NI to 2.6: Was there potential for a substantial impact (on the result) of the failure to analyse participants in the group to which they were randomized?	NA	
	**Risk of bias judgement**	**Low**	
Bias due to missing outcome data	3.1 Were data for this outcome available for all, or nearly all, participants randomized?	PN	12.5% dropped out. 3 dropped from the PT and 2 from the ST group. Mainly nn-intervention related.
	3.2 If N/PN/NI to 3.1: Is there evidence that result was not biased by missing outcome data?	PN	No they did not perform any imputation or sensitivty analysis.
	3.3 If N/PN to 3.2: Could missingness in the outcome depend on its true value?	PN	The drops outs were related to "non-intervention health related" and two due to professional issues
	3.4 If Y/PY/NI to 3.3: Is it likely that missingness in the outcome depended on its true value?	NA	
	**Risk of bias judgement**	**Low**	
Bias in measurement of the outcome	4.1 Was the method of measuring the outcome inappropriate?	N	
	4.2 Could measurement or ascertainment of the outcome have differed between intervention groups?	PN	
	4.3 Were outcome assessors aware of the intervention received by study participants?	N	"Tests were performed by the same investigators (who were blinded regarding group allocation) before the intervention and 8 and 16 weeks post-intervention."
	4.4 If Y/PY/NI to 4.3: Could assessment of the outcome have been influenced by knowledge of intervention received?	NA	
	4.5 If Y/PY/NI to 4.4: Is it likely that assessment of the outcome was influenced by knowledge of intervention received?	NA	
	**Risk of bias judgement**	**Low**	
Bias in selection of the reported result	5.1 Were the data that produced this result analysed in accordance with a pre-specified analysis plan that was finalized before unblinded outcome data were available for analysis?	NI	No preregistration reported
	5.2 ... multiple eligible outcome measurements (e.g. scales, definitions, time points) within the outcome domain?	PN	Standard scales used at one time point. Reported functional outcomes. For ex, get up and go required for meta. Reported post scores
	5.3 ... multiple eligible analyses of the data?	PN	Yes. But post data reported.
	**Risk of bias judgement**	**Low**	
**Overall bias**	**Risk of bias judgement**	**Low**	

**Unique ID**	13	**Study ID**	Monteiro	**Assessor**	
**Ref or Label**	Monteiro	**Aim**	assignment to intervention (the 'intention-to-treat' effect)		
**Experimental**	Power	**Comparator**	Control	**Source**	
**Outcome**	Physical Function	**Results**		**Weight**	1

Domain	Signalling question	Response	Comments
Bias arising from the randomization process	1.1 Was the allocation sequence random?	PY	No info about method of randomization or concealment.
	1.2 Was the allocation sequence concealed until participants were enrolled and assigned to interventions?	NI	
	1.3 Did baseline differences between intervention groups suggest a problem with the randomization process?	PN	No imbalances are apparent or observed imbalances are compatible with chance.
	**Risk of bias judgement**	**Some concerns**	
Bias due to deviations from intended interventions	2.1.Were participants aware of their assigned intervention during the trial?	Y	
	2.2.Were carers and people delivering the interventions aware of participants' assigned intervention during the trial?	Y	
	2.3. If Y/PY/NI to 2.1 or 2.2: Were there deviations from the intended intervention that arose because of the experimental context?	PN	The control group was exercising too. They were exercising at a center. Deviations due to trial context are very unlikely
	2.4 If Y/PY to 2.3: Were these deviations likely to have affected the outcome?	NA	
	2.5. If Y/PY/NI to 2.4: Were these deviations from intended intervention balanced between groups?	NA	
	2.6 Was an appropriate analysis used to estimate the effect of assignment to intervention?	PN	Yes. They did not use an ITT. The researchers excluded partipants with < 85% attendance. But no info about how many were excluded and from which groups.
	2.7 If N/PN/NI to 2.6: Was there potential for a substantial impact (on the result) of the failure to analyse participants in the group to which they were randomized?	PY	
	**Risk of bias judgement**	**High**	
Bias due to missing outcome data	3.1 Were data for this outcome available for all, or nearly all, participants randomized?	NI	No info about drop outs or missing data. All partcipants completed the 8 month study it appears.
	3.2 If N/PN/NI to 3.1: Is there evidence that result was not biased by missing outcome data?	PN	
	3.3 If N/PN to 3.2: Could missingness in the outcome depend on its true value?	NI	There was no info to evaluate this info. Author emailed 10 subjects dropped

© 2022 Balachandran AT et al. *JAMA Network Open*.

	3.4 If Y/PY/NI to 3.3: Is it likely that missingness in the outcome depended on its true value?	NI	(25% missing), but no info on the groups from which they dropped.
	**Risk of bias judgement**	**Some concerns**	
**Bias in measurement of the outcome**	4.1 Was the method of measuring the outcome inappropriate?	PN	Had validated and sensitive measures.
	4.2 Could measurement or ascertainment of the outcome have differed between intervention groups?	PN	Outcome measurement (data collection) involve the same measurement methods and thresholds, used at comparable time points
	4.3 Were outcome assessors aware of the intervention received by study participants?	N	No information about blinding. But authors email confirmed blinding. Assessors were blinded
	4.4 If Y/PY/NI to 4.3: Could assessment of the outcome have been influenced by knowledge of intervention received?	NA	
	4.5 If Y/PY/NI to 4.4: Is it likely that assessment of the outcome was influenced by knowledge of intervention received?	NA	
	**Risk of bias judgement**	**Low**	
**Bias in selection of the reported result**	5.1 Were the data that produced this result analysed in accordance with a pre-specified analysis plan that was finalized before unblinded outcome data were available for analysis?	NI	No pre-specified analysis or protocol mentioned
	5.2 ... multiple eligible outcome measurements (e.g. scales, definitions, time points) within the outcome domain?	PN	Standard scales used at one time point. Reported functional outcomes. For ex, chair stand and get up and go required for meta.
	5.3 ... multiple eligible analyses of the data?	PN	Yes. But post data reported.
	**Risk of bias judgement**	**Low**	
**Overall bias**	**Risk of bias judgement**	**High**	

© 2022 Balachandran AT et al. *JAMA Network Open*.

© 2022 Balachandran AT et al. *JAMA Network Open*.
